# Impact of GPT-4–Generated Discharge Letters on Patients’ Medical Comprehension: Prospective Crossover Study

**DOI:** 10.2196/81243

**Published:** 2026-02-26

**Authors:** Friederike Holderried, Alessandra Sonanini, Christian Stegemann–Philipps, Anne Herrmann–Werner, Philipp Spitzer, Martina Guthoff, Nils Heyne, Konstantin Sering, Martin Holderried, Felix Eisinger

**Affiliations:** 1 Tübingen Institute for Medical Education (TIME) University of Tübingen Tübingen Germany; 2 Department of Psychiatry and Psychotherapy University of Erlangen-Nürnberg Erlangen Germany; 3 Nephrological Center Villingen-Schwenningen Villingen-Schwenningen Germany; 4 Department of Diabetology, Endocrinology, Nephrology University of Tübingen Tübingen Germany; 5 Karlsruhe Municipal Hospital Karlsruhe Germany

**Keywords:** artificial intelligence, GPT-4, health care communication, large language models, patient education, patient letters, patient safety

## Abstract

**Background:**

Patients often struggle to understand standard hospital discharge letters, increasing the risk of medication errors and misunderstandings. According to cognitive load theory (CLT), complex, information-dense texts can overload working memory and impair comprehension. Artificial intelligence tools that generate patient-centered versions could help reduce extraneous cognitive load and bridge this gap. However, evidence for their effectiveness remains limited.

**Objective:**

This study aimed to evaluate whether GPT-4 (OpenAI)–generated patient-centered letters improve standardized patients’ retention and understanding of safety-relevant medical information compared with standard hospital discharge letters, and to explore potential effects on cognitive load as described by CLT.

**Methods:**

In this prospective, randomized, crossover study, 48 trained standardized patients received a conventional discharge letter for an assigned disease (out of 3) and its matching GPT-4–generated patient-centered letter. Participants read one version first, identified predefined safety-relevant “learning objectives,” and then repeated the task with the alternate version. The primary outcome was the proportion of learning objectives fully, partially, or not reported. In a secondary analysis, results were stratified by content field (Medication, Organization, Prevention of Complications, Lifestyle/Disease Management) and Bloom taxonomy level (“Remember,” “Understand”).

**Results:**

The letter type significantly influenced comprehension (odds ratio [OR] 1.74, 95% CI 1.45-2.08; *P*<.001). Patient letters, compared with discharge letters, led to higher rates of fully (490/1073, 45.7% vs 413/1073, 38.5%) or partially (322/1073, 30% vs 287/1073, 26.7%) stated learning objectives and fewer omissions (261/1073, 24.3% vs 373/1073, 34.8%). Participants performed better on “Remember” than on “Understand” learning objectives, regardless of letter type (OR 3.33, 95% CI 1.96-5.88; *P*<.001). Compared with standard hospital discharge letters, patient letters consistently improved results at both cognitive levels (“Remember”: 278/545, 51% vs 242/545, 44.4%; “Understand”: 212/528, 40.2% vs 171/528, 32.4% fully stated). The effect of patient letters varied by content field (*P*<.001). The greatest improvements were observed for “Medication” (170/254, 66.9% vs 129/254, 50.8% fully stated) and “Organization” (78/158, 49.4% vs 62/158, 39.2% fully stated). Improvements in the content field “Prevention of Complications” were modest, and those for “Lifestyle/Disease Management” were even smaller across all conditions. A total of 24.3% (261/1073) of key information remained unrecognized.

**Conclusions:**

In this explanatory study, GPT-4–generated patient letters improved comprehension of safety-relevant discharge information among standardized patients, particularly regarding medication and organizational aspects. However, they were less effective in supporting higher-order understanding, such as risk prevention or lifestyle management. These hypothesis-driven findings can be interpreted within a CLT framework and may motivate prospective evaluation of multimodal, iterative supports.

## Introduction

The transition from inpatient to outpatient care represents a critical juncture for information exchange and patient safety, often marked by communication errors and incomplete understanding [1]. Nearly half (49%) of patients experience at least 1 postdischarge medical error, most frequently concerning medication management, diagnostic workup, or test result follow-up [2]. Effective patient understanding during this phase is crucial, as misinterpretation or omission of medical instructions can directly impact adherence, follow-up care, and safety outcomes [3,4]. Studies estimate that up to 78% of patients misunderstand key elements of their hospital care and discharge instructions [5], and approximately half (50.8%) of patients receiving polypharmacy misinterpret at least 1 dosage instruction [6].

Patients’ comprehension of discharge information is influenced by multiple factors, including health literacy, educational background, prior knowledge, and emotional stress during this vulnerable phase [7,8]. Considering that 36% of US adults have limited health literacy [9], a substantial proportion of patients may struggle to fully understand and follow postdischarge instructions, which are typically written for communication between health care professionals and often fail to meet patients’ informational needs [10]. Therefore, there is a clear need for discharge documents that are patient-centered, clearly structured, and accessible; these will be referred to as patient letters throughout this manuscript.

Recent advances in artificial intelligence (AI), particularly large language models (LLMs) such as GPT-4 (OpenAI), offer a promising approach to address this need [11]. By transforming complex medical documents into plain-language patient letters, AI has the potential to improve patients’ comprehension and recall of important medical information. Several feasibility studies have demonstrated that LLMs can generate readable and accurate patient letters from standard discharge documents, highlighting their technical capability to simplify language, structure information, and provide consistent formatting [12-14]. To date, only a small number of studies have investigated the impact of GPT-generated patient letters on patient understanding [15-17]. These studies have primarily relied on semistructured interviews or multiple-choice assessments, offering only quantitative evaluations of patient knowledge. A more nuanced understanding of patient comprehension is still lacking, particularly regarding which types of information are retained, how the cognitive complexity of content influences understanding, and whether comprehension differs across medical content domains. Accordingly, evidence on the educational impact and cognitive benefits of AI-generated patient communications remains limited.

This study is based on our previous work, in which we explored the feasibility of transforming discharge letters into patient letters using GPT-4 [12]. That study primarily focused on the accuracy of the generated letters and measures of patient-centeredness, demonstrating that overall, GPT-4 can produce readable and structured patient-directed documents, with some limitations including omissions. Building on these findings, this study shifts the focus to an application-oriented perspective. It investigates whether GPT-4–generated patient letters improve comprehension, using standardized patients (SPs) to simulate real-world understanding and follow-up tasks.

To systematically evaluate comprehension, we framed patient understanding as a learning process informed by cognitive load theory (CLT) [18]. According to CLT, learning efficiency depends on three types of cognitive load:

1. Intrinsic load refers to the inherent complexity of the information and is dependent on the learner’s level of expertise. In the context of discharge letters, intrinsic load is generally high due to complex medical content and procedural details, and it can only be modified to a limited extent.
2. Extraneous load reflects mental resources allocated to elements that do not contribute to learning or schema acquisition. High extraneous load can arise from poorly structured information, excessive jargon, or frequent use of abbreviations [18]. In our prior study, we demonstrated that GPT-4–generated patient letters mitigated these contributing factors by providing increased repetition of key points, fewer abbreviations, and reduced use of medical terminology [12].
3. Germane load represents the mental resources devoted to acquiring and automating schemata in long-term memory. In our context, the goal is to maximize germane load so that patients allocate as much cognitive energy as possible to truly understand, retain, and integrate the medical information.

Guided by this theoretical framework, the study addressed the following research questions:

- Does the reduction of extraneous load through AI-generated patient letters translate into improved comprehension of safety-relevant information?
- Are comprehension gains consistent across different content domains (eg, Medication, Organization, Prevention of Complications, and Lifestyle)?
- Do comprehension improvements differ between lower-order (“Remember”) and higher-order (“Understand”) cognitive processes as defined by Bloom taxonomy [19]?

By combining a patient-centered perspective with a learning-theoretical framework, this study examines the educational potential and limitations of AI-generated patient letters and considers how they might contribute to patient safety during vulnerable care transitions.

## Methods

### Study Design and Setting

In our previous study, we developed a methodology for generating GPT-4–based patient letters, demonstrating the feasibility of transforming discharge letters into patient-friendly formats while maintaining medical accuracy and patient-centeredness. The prompting strategy and human review process were described in detail in that publication [12].

Building directly on this work, this prospective, randomized, crossover study with SPs used 3 discharge letters and the corresponding GPT-4–generated patient letters from our prior study, each representing a common chronic condition: arterial hypertension, type 2 diabetes mellitus, and diabetic kidney disease. These diagnoses were selected because they are highly prevalent and frequently occur in both inpatient and outpatient care. The letters are provided in Multimedia Appendix 1 in German (original) and English (translated).

**Figure 1:**
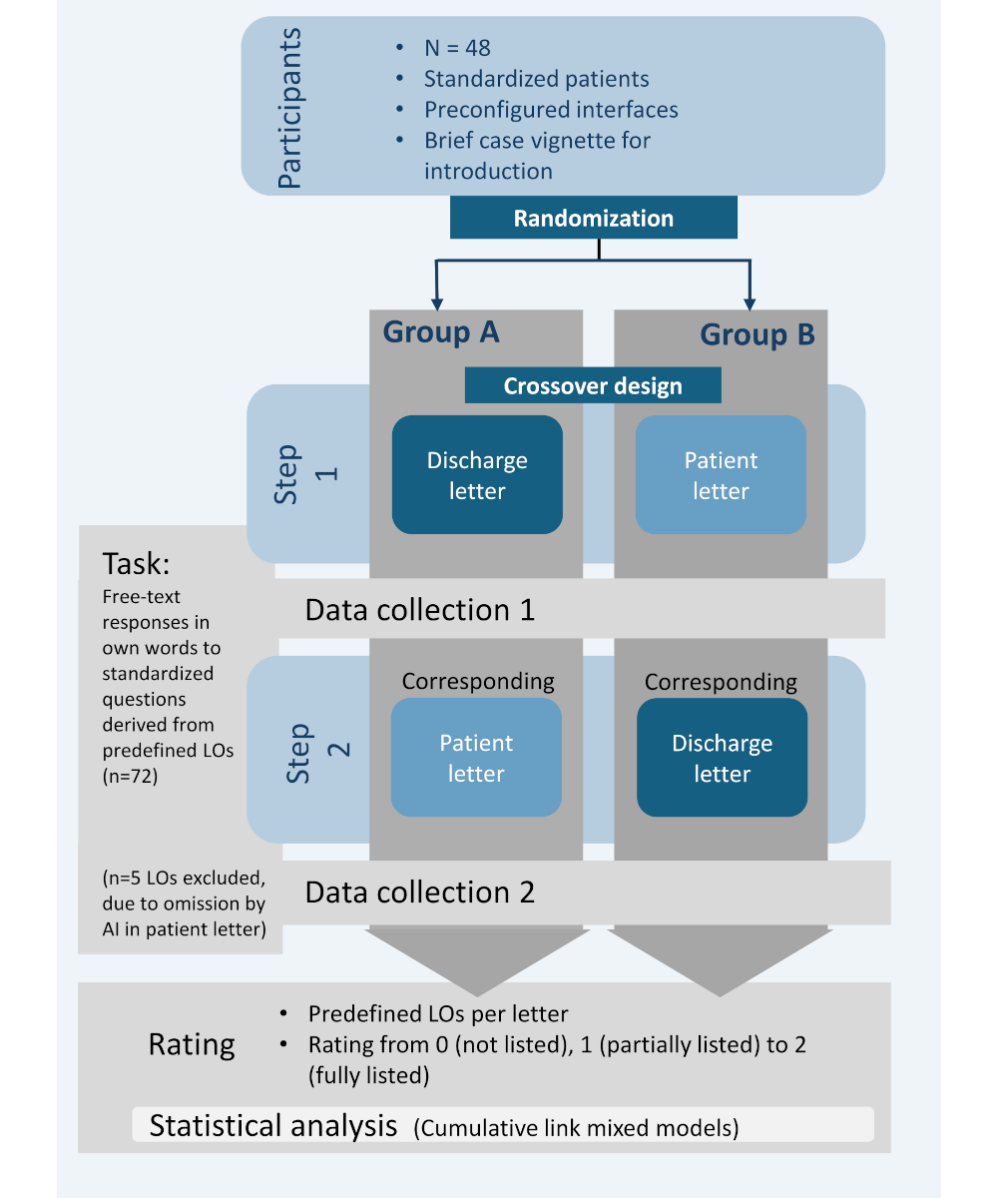
Overview of study design and rating procedure in a prospective, randomized crossover study comparing the retention of predefined learning objectives by standardized patients after reading either a standard hospital discharge letter or a GPT-4–generated patient letter. AI: artificial intelligence; LO: learning objectives.

Both letter formats were presented to 48 SPs using a crossover design (Figure 1). We compared perception and retention of key medical information—defined as “learning objectives”—presented in the letters. 

### Participants and Study Procedures

A total of 48 SPs participated in our study (Multimedia Appendix 2). SPs are individuals who are specifically trained as patient actors to portray medical patients in a consistent and realistic manner, and they participated in the study in their specific role [20,21]. The sample size was determined by the number of participants available for recruitment and was not calculated prior to the study. The SPs were recruited from the SP programs of the Universities of Tübingen and Erlangen, Germany. Data were collected between May and September 2024. After reading a brief case vignette covering basic medical and socioeconomic information, each participant was randomized to the discharge letter (group A) or its patient-friendly version (group B). Participants were not informed about the sequence in which they would receive the letters. However, the differences between the discharge letter and the GPT-4–generated patient letter were apparent, making full blinding impossible. Therefore, no formal assessment of blinding adequacy was performed.

At both sites, letters were presented digitally through a web-based interface. Participants were instructed to read each letter with care and were permitted to scroll back to the text while answering the open-ended questions. This setting mirrors the real-world access to written discharge information. The SPs extracted key information from 4 content fields and answered predefined open questions (Multimedia Appendix 3). This approach was chosen because continuous access limits short-term memory bias and mirrors real practice [22]. To foster active knowledge construction, participants were instructed to answer in their own words without copying. Letters were then crossed over, and the task was repeated with the alternate version.

### Learning Objectives and Content Fields

For each of the 3 clinical cases (arterial hypertension, diabetes mellitus, and diabetic kidney disease), we defined 24 safety-related learning objectives that cover information considered essential by the treating physicians for patient self-management after discharge (eg, knowledge of medication changes, warning symptoms, and follow-up appointments). The learning objectives were designed and revised by an interdisciplinary committee of 4 physicians (specialized in internal medicine, nephrology, diabetology, general medicine, and medical education) based on current clinical guidelines [23-27] and their professional experience. They were formulated as short, observable statements (eg, “The patient can ... list” and “The patient understands that ...”) and checked for comprehensibility and redundancy.

For each case, the 24 learning objectives were evenly distributed across the levels of the revised Bloom taxonomy, with 12 learning objectives in the “Remember” category and 12 in the “Understand” category. In Bloom taxonomy, the “Remember” category refers to the ability to recall facts and basic concepts, while the “Understand” category goes beyond recall and involves interpreting, explaining, and making sense of the information [19,28]. Each learning objective was further mapped to 1 of 4 content fields: “Organization” (follow-up tasks), “Medication” (drug changes), “Prevention of Complications” (early detection and management of disease-related complications), and “Lifestyle/Disease Management” (self-care advice/lifestyle modifications; Multimedia Appendix 3). This structure ensured comparable cognitive demands and content coverage across all 3 cases and enabled stratified analyses by content area and Bloom category. The process of defining learning objectives and creating the original discharge letters has been described in detail in our previous publication [12].

Across the 3 letters used in this study, 72 learning objectives were assessed in total (24 per letter). A total of 5 of these 72 learning objectives were not represented in the GPT-4–generated patient letters because the model did not carry them over from the original discharge letters. These learning objectives were therefore excluded from the analysis for both letter types (Multimedia Appendix 4). The quality and completeness of the translation from discharge letter to patient letter were examined in detail in our previous publication and were therefore not reassessed within the scope of this study [12]. After this exclusion, 34 of the 67 remaining learning objectives were classified as “Remember” and 33 as “Understand” according to Bloom taxonomy (Multimedia Appendix 3).

### Assessment Approach

Reading the discharge or patient letter and then answering the case questions was designed as a structured learning assessment sequence [29]: the letters provided the information to be learned, and the SPs’ responses reflected what they had actually understood. To capture constructed understanding rather than simple recognition, we used open-ended questions that required participants to formulate their answers in their own words rather than choosing from predefined options.

Open-ended response formats are less prone to guessing than multiple-choice questions and are better suited for assessing higher levels of processing for learning objectives at the “Understand” level. This approach closely aligns the assessment with the predefined learning objectives and the intended depth of understanding. This choice is supported by assessment literature [30].

### Outcome Measures

The primary outcome was the understanding of safety-related information at the level of individual learning objectives. Each participant response (participant × learning objective × letter) was rated on an ordinal scale with 3 categories reflecting the degree of goal achievement in the open-ended response (0=not specified, 1=partially specified, 2=fully specified). The main comparison of interest was the difference between the patient letters generated by GPT-4 and the standard discharge letters with respect to this ordinal measure of SPs’ understanding.

Secondary end points were the distribution of this ordinal comprehension metric across the 4 predefined content areas (Organization, Medication, Prevention of Complications, and Lifestyle/Disease Management) and the distribution across the levels of Bloom taxonomy (“Remember” vs “Understand”). As an additional secondary end point, the frequency of medically incorrect answers was examined.

### Randomization

Participants at the University Hospital Tübingen were allocated to the 3 clinical cases and the order of letter presentation (discharge letter first vs patient letter first) according to a pregenerated randomization list. This list was created prior to participant recruitment using a randomly generated allocation sequence that specified both case assignment and presentation order, with approximately balanced group sizes. Participants were enrolled consecutively and assigned to the next available entry on the randomization list. Allocation was not known in advance to either participants or study personnel conducting the sessions, thereby preserving allocation concealment at the participant level.

Participants from the University of Erlangen, who took part remotely via a web-based study platform, were randomized using a computer-generated 1:1 allocation algorithm implemented within the platform. Upon enrollment, the platform automatically assigned participants to the letter-order sequence and the clinical case.

The use of 2 different randomization methods resulted from the different study sites: on-site participation in Tübingen required an offline procedure, whereas the Erlangen cohort participated exclusively online. Randomization was conducted separately within each site and not across sites. Although randomization procedures differed between sites, both approaches ensured allocation concealment at the individual level. Importantly, the substantive data collection procedure (including exposure to the letters and assessment of learning objectives) was identical at both locations.

### Data Analysis

#### Rating Process

The answers of the SPs were independently rated by 2 experienced clinicians (FH and AS) to determine whether the predefined learning objectives were correctly and completely identified and reported. The rating process followed a predefined, ordinally scaled rating structure, ranging from “not listed” over “partially listed” to “fully listed,” and disagreements were solved by discussion. A specific definition was established in advance for each learning objective (Multimedia Appendix 3). Raters were blinded to both the letter type (discharge letter or patient letter) and the order in which it was presented. No formal assessment of blinding adequacy was performed. The raters achieved a Cohen κ of 0.97. Due to the high level of agreement, one of the two ratings was selected at random for use in the statistical analysis.

#### Statistical Analysis

Due to the ordinal nature of the ratings of the learning objectives (comprehensively reported, partially reported, and not reported), cumulative link mixed models were fitted to the data. Random intercepts for participant and learning objective were used to account for participant and learning objective random effects (Multimedia Appendix 5). The fixed effects of letter type, Bloom category, and content field were investigated as variables of interest with model comparisons. The fixed effects disease type and time point of measurement were included as controls. Relevant models were assessed, and alternatives were considered. *P* values for differences in individual content fields were calculated by subsetting the data to each content field. Models were compared according to the Akaike Information Criterion and likelihood ratio tests. No missing data were present. The analyses were conducted using R software (version 4.4; R Foundation for Statistical Computing) and the ordinal package version 2023.12-4.1 [31,32].

#### Reporting Standards

The reporting of this study followed the STROBE (Strengthening the Reporting of Observational Studies in Epidemiology) guideline for cohort studies [33]. A reporting checklist can be found in the Multimedia Appendix 6.

### Ethical Considerations

This study was approved by the local ethics committee (778/2023BO2) of Tübingen University Hospital, Germany. Written informed consent was obtained from the SPs before inclusion in the study.

No direct identifiers (eg, names or dates of birth) were collected in the study. A short demographic questionnaire (age, gender, educational background, and prior experience as an SP) was completed by the SPs; these results were stored separately and not linked to individual response data. During data collection, response data were temporarily saved on secured cloud infrastructure; evaluation data and the separate demographic file were stored in different locations with a role-based access control. Reidentification was not possible at any time. Due to data loss at one site, demographics were available for 38 out of 48 SPs. As demographics were not used in the analysis of learning objective performance, and there is no evidence of systematic bias between sites, this missing information does not affect the validity of the study outcomes. The study was conducted in accordance with the Declaration of Helsinki.

## Results

### Demographic Data

The median age of the participants was 46.7 (IQR 28.3-65.0) years, with a female-to-male ratio of approximately 1.92:1. The majority (24/38, 63.2%) had a university degree. Task order did not influence performance (*P*=.80), although participants performed slightly better on their second attempt (odds ratio [OR] 1.23, 95% CI 1.03-1.47; *P*=.02). The type of disease did not have a significant main effect on the retention rate of learning objectives (*P*=.96; Multimedia Appendix 5). Therefore, all patient data were pooled for the subsequent comparison of the discharge letter and the patient letter.

### Patient Letters vs Discharge Letters

**Figure 2:**
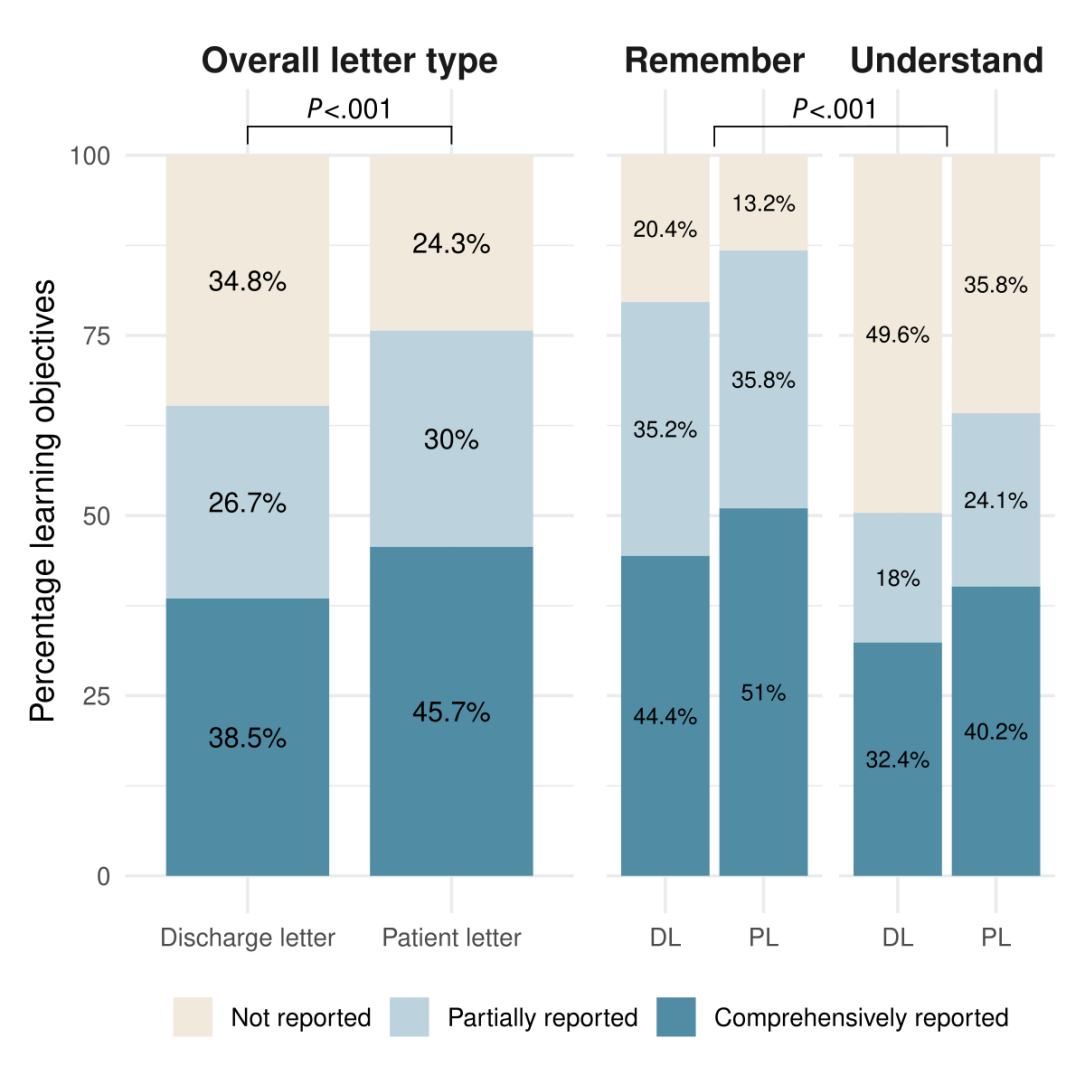
Percentage of predefined learning objectives that were comprehensively reported, partially reported, or not reported by standardized patients after reading either a standard hospital discharge letter (DL) or a GPT-4–generated patient letter (PL). Results are shown overall (left) and separately (right) for the Bloom taxonomy levels “Remember” and “Understand.” Statistical comparison was performed using cumulative link mixed models. Overall, standardized patients retained significantly more learning objectives with the patient letter than with the discharge letter. Across both letter types, performance was significantly higher for “Remember” than for “Understand” learning objectives.

**Figure 3:**
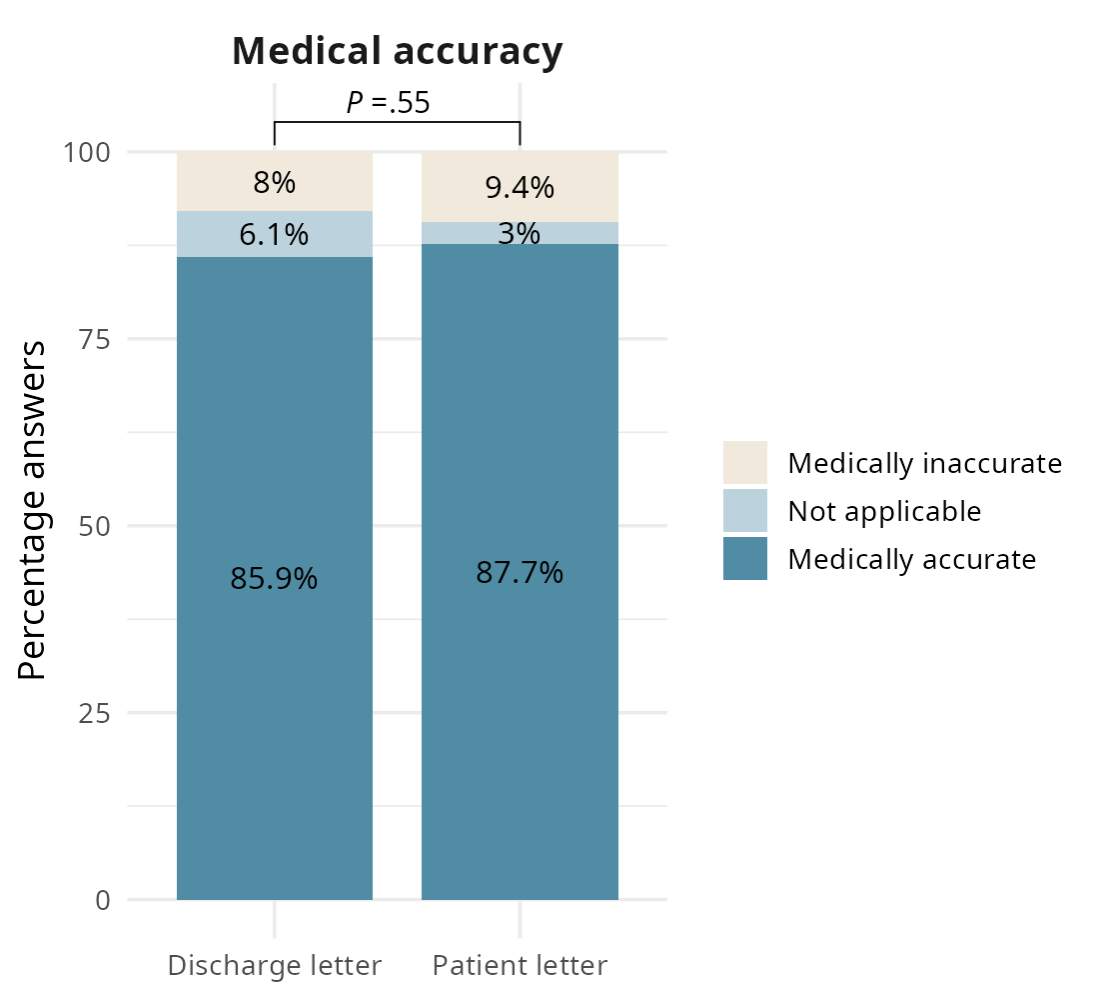
Medical correctness of standardized patient responses after reading either a standard hospital discharge letter or a GPT-4–generated patient letter. The figure illustrates the percentage of responses rated as medically accurate, medically inaccurate, or not applicable (statements without medical content). Statistical comparison was conducted using cumulative link mixed models.

Overall, patient letters improved information uptake: SPs retained more learning objectives after reading the patient letter compared with the discharge letter (OR 1.74, 95% CI 1.45-2.08; *P*<.001; Figure 2). Comprehensive reporting rose to 45.7% (490/1073) with the patient letter compared to 38.5% (413/1073) with the discharge letter. Partial reporting also improved (322/1073, 30% vs 287/1073, 26.7%), while omissions decreased from 34.8% (373/1073) to 24.3% (261/1073; Figure 2; Multimedia Appendix 7). Remarkably, despite overall gains, those 24.3% (261/1073) of all ratings remained “not reported” after reading the patient letter. Because omissions varied by participant, we conducted an item-level analysis to identify objectives consistently missed (predefined as “not reported” in ≥50% of the cases). A total of 6 (9%) of 67 objectives met this criterion. By Bloom level, 5 out of 6 were “Understand” and 1/6 “Remember”; by content field, 3 out of 6 fell under “Prevention of Complications,” 2 out of 6 under “Lifestyle/Disease Management,” and 1 out of 6 under “Organization” (Multimedia Appendix 8). On the other hand, the overall proportion of medically incorrect reports by the participants was low, with no significant difference between the letter types (*P*=.55; Figure 3).

### Impact of Cognitive Demand (Bloom Taxonomy) on Information Uptake

A subgroup analysis of learning objectives based on Bloom taxonomy revealed that, regardless of letter type, participants performed better on learning objectives classified under the “Remember” category compared to those classified under the “Understand” category (OR 3.33, 95% CI 1.96-5.88; *P*<.001; Figure 2). In both categories, the proportion of comprehensively reported learning objectives was higher when presented in the patient letter format compared to the discharge letter format (278/545, 51% vs 242/545, 44.4% for “Remember” and 212/528, 40.2% vs 171/528, 32.4% for “Understand”; Figure 2; Multimedia Appendix 7). A subsequent analysis showed no significant interaction between Bloom taxonomy level and letter type (*P*=.13), indicating that the observed benefits of the patient letter were consistent across both cognitive levels.

### Impact of Content Field on Information Uptake

In a subgroup analysis based on content fields, we observed a statistically significant interaction effect between the type of letter and the content field (*P*<.001), indicating that the benefit of the patient letter depends on the content field. Additional values for effect size, OR, and CI for the results in this paragraph are illustrated in Multimedia Appendix 9. A substantial increase in comprehension associated with the patient letter was observed for the domain of “Medication” (*P*<.001), with a rate of accurately reported learning objectives of 66.9% (170/254) compared to 50.8% (129/254) for the discharge letter. In the GPT-4–generated patient letter, the changes and purpose of each drug were explicitly stated in plain language (eg, “You were also given two other medications, metformin and empagliflozin, which help control blood sugar”), whereas the standard discharge letter contained this information in a condensed table format without further explanation. For further details on the translation process from discharge to patient letters, we refer to our previous work [12].

**Figure 4:**
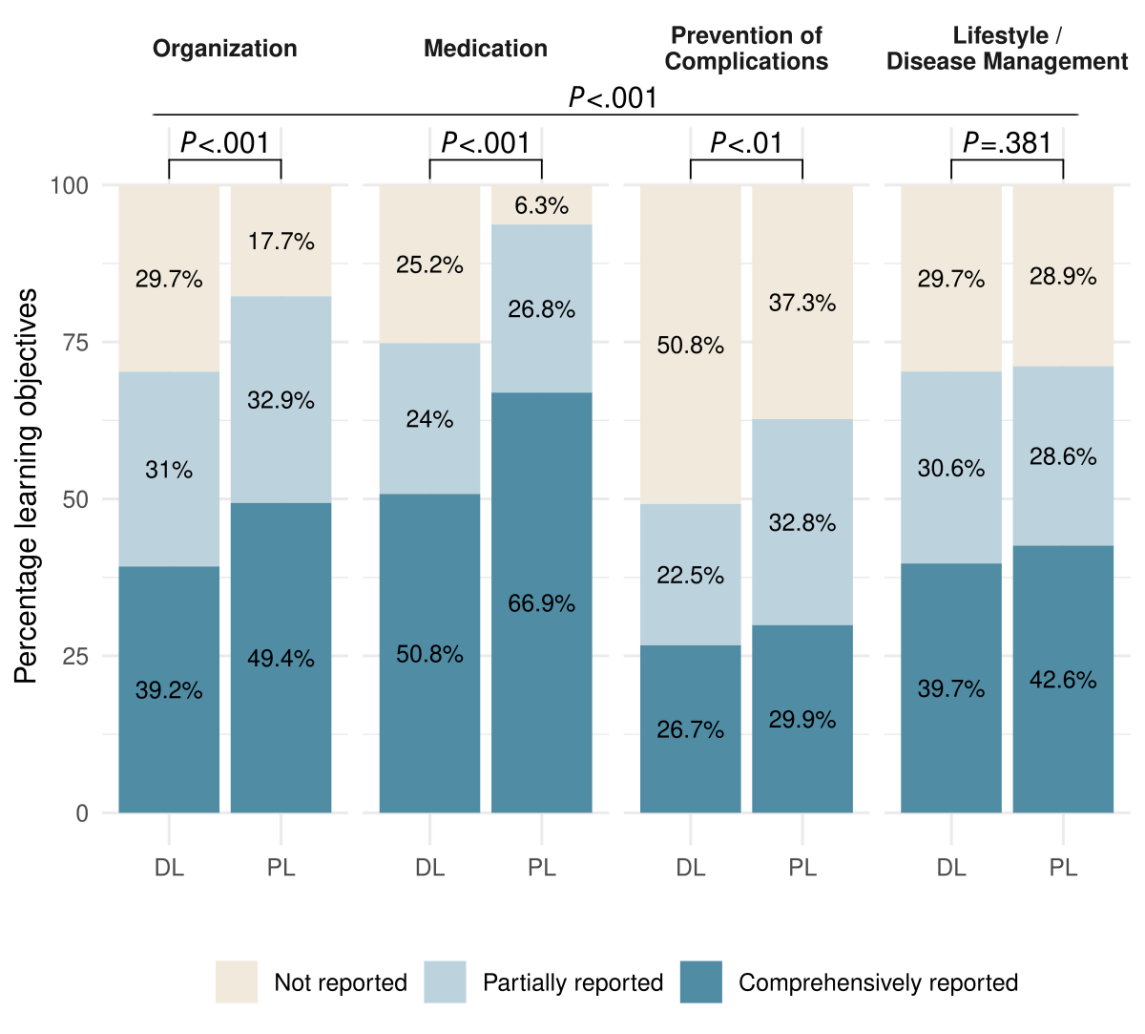
Percentage of predefined learning objectives that were comprehensively reported, partially reported, or not reported by standardized patients after reading either a standard hospital discharge letter (DL) or a GPT-4–generated patient letter (PL). Results are displayed across 4 different content categories: Organization, Medication, Prevention of Complications, and Lifestyle/Disease Management. Differences between letter types were evaluated using cumulative link mixed models. Significant improvements were achieved with the patient letter in the content fields “Medication” and “Organization,” whereas gains were modest for “Prevention of Complications” and minimal for “Lifestyle/Disease Management.”.

The second largest improvement was seen in the domain of “Organization” (78/158, 49.4% vs 62/158, 39.2%; *P*<.001; Figure 4; Multimedia Appendix 7). In contrast, improvements observed with the patient letter in the content fields “Lifestyle/Disease Management” (*P*=.38) and “Prevention of Complications” (*P*<.01) were small and medium. In the content field “Prevention of Complications,” the patient letter resulted in a minor increase in the proportion of partially addressed learning objectives compared to the discharge letter (102/311, 32.8% vs 70/311, 22.5%). However, the rate of completely reported learning objectives remained the lowest compared to the other content fields (Figure 4; Multimedia Appendix 7). Regarding the content field “Lifestyle/Disease Management,” there was even a small decrease observed for the partially reported learning objectives (100/350, 28.6% in the patient letter vs 107/350, 30.6% in the discharge letter). However, the amount of correctly stated learning objectives still showed a small increase (149/350, 42.6% in the patient letter vs 139/350, 39.7% in the discharge letter; Figure 4; Multimedia Appendix 7).

## Discussion

### Principal Findings

This prospective, explanatory study with SPs showed that AI-generated patient letters increased the retrieval of medication-related learning objectives to 66.9% (170/254) and organizational objectives to 49.4% (78/158), compared with 50.8% (129/254) and 39.2% (62/158), respectively, after reading the standard hospital discharge letters. These gains are larger than those reported in earlier single-arm or pre-post studies of human or AI-generated patient letters that lacked counterbalancing [13,34,35]. By scoring open, free-text answers, we also captured a broader slice of comprehension than recent GPT-based studies that relied on recognition formats [15-17]. However, the reliance on predefined learning objectives, evaluated by clinicians, may still miss nuances of SP understanding. In particular, misconceptions and emotional interpretations may be underreported, and these remain important aspects for future qualitative work.

### Content- and Bloom-Specific Insights

Although overall performance improved, SPs still failed to report, on average, nearly a quarter of the key information, highlighting a persistent patient safety gap for patients. Gains clustered in the areas of Medication and Organization, which are long-recognized weak spots: <50% of patients understand their discharge medicines [36], and medication errors account for roughly half of preventable harm [37]. By increasing the retention of medication-related information, we hypothesize that patient letters have the potential to improve medication management and reduce medication errors, thereby potentially contributing to enhanced patient safety. Similarly, improving awareness of necessary follow-up appointments may also support patient safety by reducing missed visits and associated complications. Previous research supports these mechanisms: written patient information material has been shown to increase medication adherence [38], missed follow-up appointments predict readmissions and emergency visits [39-41], and low health literacy is associated with higher readmission rates [42]. Moreover, communication interventions at hospital discharge are significantly linked to fewer readmissions and higher treatment adherence [4]. While these findings provide a rationale for the use of AI-generated patient letters, the effects on actual patient safety outcomes remain to be empirically evaluated in future research.

In contrast, improvements in the content field “Prevention of Complications” were modest, and those for “Lifestyle/Disease Management” were even smaller. Notably, 37.3% (116/311) of the key information regarding “Prevention of Complications” remained unknown. While higher cognitive demand likely contributed, other factors such as complex sentence structures, domain-specific phrasing, multistep reasoning, and the absence of visual aids may have further limited comprehension. As shown in our previous study, GPT-4 also faced greater challenges translating “Prevention of Complications” content compared with other domains [12]. Interestingly, although GPT-4 more successfully simplified “Lifestyle/Disease Management” information, SP performance remained low, suggesting that simplification alone does not guarantee comprehension when integration into practical self-management is required, and suggesting a distinction between content that is challenging for the AI to translate versus content that is difficult for SPs to understand and apply. Stratifying learning objectives by Bloom taxonomy showed higher performance for “Remember” than for “Understand” items (278/545, 51% vs 212/528, 40.2% correct with the patient letter), and 5 out of 6 objectives omitted by ≥50% of participants fell into the “Understand” category. This difference is unsurprising given that “Remember” tasks primarily involve locating explicit information, whereas “Understand” tasks require higher-order cognitive processes. Importantly, the patient letter improved performance in both categories compared with the discharge letter (“Remember”: 278/545, 51% vs 242/545, 44.4%; Understand: 212/528, 40.2% vs 171/528, 32.4%), and there was no significant interaction between letter type and Bloom level (*P*=.13), indicating that the observed benefit of the patient letter was approximately equal for both cognitive levels.

These findings suggest that GPT-4–generated patient letters can support not only the retrieval of factual information but may also modestly enhance comprehension-oriented learning objectives, though higher-level understanding remains more challenging. This is consistent with prior longitudinal research demonstrating that discharge instructions decay rapidly without reinforcement [43-45].

The clustering of consistently missed objectives in “Prevention of Complications” and, secondarily, “Lifestyle/Disease Management,” together with the predominance of “Understand”-level items, suggests that a static, text-only letter may not be sufficient in contexts where patients need to link symptoms to actions, reason about conditional risks (eg, sleep apnea as source of high blood pressure), or maintain self-management routines (eg, home blood pressure documentation or dietary change).

### Didactic Implications (CLT)

CLT provides a helpful framework for interpreting our findings [18]. Recent work applying CLT to clinical practice shows that stress, emotion, and uncertainty increase intrinsic cognitive load and that working memory depletion can impair clinical performance [46]. Against this background, it seems plausible that linguistic simplification and better structure primarily reduce extraneous cognitive load. Our prior work demonstrated that GPT-4 can reorganize discharge letters into patient letters mainly by removing jargon and chunking information [12], which likely supports this reduction. While discharge letters are generally already well-structured, AI-generated patient letters further optimize content organization in a patient-centered manner.

We did not directly measure intrinsic, extraneous, or germane cognitive load in this study; CLT is therefore used as an interpretive framework rather than an empirically tested mechanism. Our learning objectives were deliberately designed so that each content field contained both “Remember” and “Understand” objectives. However, objectives that remained most difficult (defined as those omitted by ≥50% of participants) were predominantly from the “Understand” category in the area of “Prevention of Complications” and, to a lesser extent, in the area of “Lifestyle/Disease Management.” These learning objectives (eg, hypoglycemia symptom-action mapping, recognition of statin-associated muscle symptoms, dehydration-related kidney risk, or the contribution of home blood pressure monitoring and Mediterranean diet to long-term disease control) require the integration of multiple information elements, conditional if-then thinking, and sustained self-regulation. Within the framework of CLT, they can therefore be interpreted as tasks with a comparatively high intrinsic cognitive load.

In contrast, the largest gains from patient letters were observed predominantly in factual learning objectives in the areas of “Medication” or “Organization,” which required participants to find and reproduce explicitly stated information (eg, medication changes or appointments). Such learning objectives are likely to be particularly sensitive to a reduction in extrinsic load through simpler language and clearer structure.

Based on these findings, we conclude that AI-based translation into patient letters may reduce extraneous cognitive load, thereby possibly freeing working-memory resources for the processing of concrete, action-oriented tasks (eg, tablet strength and appointment date). However, for learning objectives with high intrinsic cognitive load, such as symptom-to-action coupling (eg, tremors-hypoglycemia compensation required), conditional risk thinking (eg, allergy present notification to the care team), or permanent self-regulation (eg, home blood pressure monitoring and dietary changes) reducing extrinsic cognitive load alone may be not sufficient to free up enough relevant resources (germane cognitive load) for deeper understanding and behavioral change.

Taken together, these CLT-based interpretations are hypothesis-generating and should be tested in future studies that directly measure cognitive load. They further suggest that patient letters may be most effective for immediate, concrete instructions, whereas higher-order learning objectives may require complementary, multimodal supports (eg, infographics, microvideos, interactive prompts, and teach-back). These, however, need prospective evaluation. Evidence for each adjunct already exists in discharge-education and adherence research [38,47,48].

Beyond CLT, factors such as stress-induced cognitive depletion, low health literacy, and limited self-efficacy may also reduce patients’ ability to process complex medical content [49-51]. Tailoring follow-up communication to these variables will be essential [52].

### Clinical Implications

The implementation of AI-generated patient letters requires careful attention to ethical, legal, and social aspects [53]. In general, human oversight (“human-in-the-loop”) remains essential when using AI to translate clinician-written discharge letters into patient letters. Our study indicates that the patient letter format alone does not yield sufficient comprehension gains for cognitively demanding learning objectives, particularly those classified as “Understand” by Bloom taxonomy and thematically related to prevention of complications or lifestyle/disease management. It is further underscored by omissions and hallucinations observed in the prior translation process [12], which required the exclusion of 5 learning objectives in this study. Therefore, establishing practical governance structures is essential for clinical practice. By identifying the specific content domains most prone to errors or omissions, our results can help to target quality-assurance efforts efficiently, making oversight both feasible and focused on the areas critical for patient safety.

From a legal perspective, robust data protection is critical, as personal patient information constitutes a central component of these letters [54,55]. Socially, simplified language may improve accessibility for patients with low health literacy and reduce language barriers [56]. While the format does not accommodate individual literacy needs or reading difficulties, it may still help reduce social barriers in discharge communication. Finally, practical and feasibility considerations include technical integration into existing clinical workflows, usability for health care staff, and costs associated with development, implementation, and maintenance. However, manual letter production is time-consuming and costly, whereas AI offers a potentially scalable solution. Effective measures without AI can be found in the literature, including patient letters written in simple language and structured summaries that reduce technical jargon and organize content for easy recall [57], portal access (facilitating review but not guaranteeing understanding) [58,59], supplementary written materials (linked with better medication adherence) [38], audio recordings of consultations (supporting recall) [43], and interactive elements such as brief teach-back or app-based prompts (which enhance encoding and memory) [47,49]. However, implementation is frequently constrained by time and workload, limited organizational support and training, workflow barriers, and gaps in digital access and use [58,59]. These problems are documented across teach-back implementation (time/sustainability barriers), discharge letter production (time pressure, authorship level, and workload effects), and portal-based delivery (digital divide: differences in access, skills, and use) [60-62].

Our study isolates the contribution of AI-generated formulations and was not designed as a head-to-head comparison. In this context, AI-generated patient letters, with clinician oversight, may offer a practical, scalable way to produce patient-centered text for domains where our data indicate clear gains (eg, medication and organization), and future work should address workflow integration, usability for clinicians, maintenance costs, and robust data protection.

### Limitations

This study has several limitations. First, we used only 1 GPT-4 version and focused on 3 chronic diseases (hypertension, diabetes, and diabetic kidney disease), which limits generalizability to other LLM versions and medical conditions. Although participants and clinician raters were blinded to letter type and order as far as possible, full blinding was not achievable, and the success of blinding was not formally assessed for either group. Because the stylistic differences between discharge letters and patient letters are recognizable, which was unavoidable given the study content, participants and raters may have inferred the format, introducing potential expectancy effects. Such effects could operate in both directions: some participants might assume the patient letter to be easier, whereas others may approach AI-generated text with skepticism, potentially reducing trust or attention. These bidirectional biases could either amplify or attenuate format-related differences. While standardized instructions, structured scoring procedures, and uniform training were used to minimize subjective influence, residual bias cannot be excluded and should be considered when interpreting the magnitude of effects. Additionally, we did not formally assess the feasibility or acceptability of patient letters through validated usability or satisfaction scales. These aspects should be addressed in future implementation studies. Furthermore, our study used standardized rather than real-world patients to examine the effects of AI-generated patient letters. This approach ensured high procedural consistency and enabled a controlled comparison between AI-generated and human-written letters, as reflected in recent high-impact AI studies using SPs [21]. However, it limits external validity; SPs are not able to fully represent real patients’ stress levels, emotional states, cognitive vulnerability, and health literacy diversity, especially in the context of acute illness or hospitalization. In clinical practice, such factors may negatively affect patient comprehension due to reduced germane cognitive load. Consequently, although the direction of the observed effects (improved understanding through AI-generated patient letters) is expected to remain, the overall level of understanding and recall would likely be lower among real patient populations for both letter types. In addition, letters were written in German and later translated for publication, limiting the generalizability of our findings to other languages. Another potential limitation of this study is the exclusion of 5 predefined learning objectives that were not present in the GPT-4–generated patient letters due to omissions, which could introduce bias in favor of the AI-generated format. However, a statistical analysis including all 72 learning objectives showed that the overall pattern of results remained unchanged despite the absence of these 5 items in the AI-generated patient letters. This suggests that the exclusion did not materially affect the observed effects. Additionally, the sample size in our study was determined by availability rather than by formal power calculation, which may limit the ability to detect subtle letter-type differences, particularly in subgroup analyses. Our findings should therefore be interpreted with caution. Finally, given the potential for hallucinations or omissions in AI-generated texts, as observed in the translation process [12], clinical review and governance are essential if AI-based tools are deployed in practice, and attention to AI transparency and the potential for unexpected outputs remains critical.

### Conclusions

This hypothesis-driven, explanatory study showed that AI-generated patient letters improved the uptake of basic, action-oriented information in SPs. This effect might reflect a reduction in extraneous cognitive load. We hypothesize that, in clinical practice, this could reduce common sources of postdischarge harm and improve patient safety. However, patient letters alone did not close gaps in higher-order understanding, particularly in risk prevention and lifestyle change. Addressing such complex learning goals may require multimodal, interactive support (eg, explanatory graphics, short videos, or app-based follow-ups [47,48,63]) that reinforce the text-based patient letter.

Future studies should therefore evaluate integrative models that layer such tools onto patient letters and tailor content to health literacy, self-efficacy, and situational resources [64], ideally within a didactic framework (eg, Observing Patient Involvement Scale or Patient Education Materials Assessment Tool [65]) to enhance actionability. Studies including more diverse medical conditions, real patient populations with varying health literacy levels, different LLMs, and multiple languages are needed to improve generalizability. We also suggest incorporating qualitative approaches in future studies (eg, think-aloud protocols or teach-back methods) to better capture nuances in patient understanding and potential contributors to medical error.

## Data Availability

The datasets generated or analyzed during this study are available from the corresponding author on reasonable request.

## Appendix

Multimedia Appendix 1 GPT-4–generated patient letters (German original and English translation).

Multimedia Appendix 2 Study flowchart. Overview of participant enrollment, randomization, crossover procedure, and analysis population.

Multimedia Appendix 3 Overview of the learning objectives, standardized questions, and the corresponding rating scheme applied to evaluate information retention and comprehension across study conditions.

Multimedia Appendix 4 List of the learning objectives that were not represented in the GPT-4–generated patient letters because the model did not carry them over from the original discharge letters. These objectives were excluded from the analysis for both letter types.

Multimedia Appendix 5 Likelihood ratio tests.

Multimedia Appendix 6 STROBE checklist detailing how each reporting item was fulfilled and where it can be found in the manuscript.

Multimedia Appendix 7 Number of learning outcomes achieved, stratified by letter type (patient letter vs discharge letter).

Multimedia Appendix 8 Item-level analysis identifying learning objectives that were consistently missed, predefined as those “not reported” by ≥50% of participants.

Multimedia Appendix 9 Odds ratios from the best-fitting cumulative link mixed model. Odds ratios represent the interaction between letter type and content field, estimated relative to an arbitrary reference category. The reference category is defined as letter type = discharge letter (DL) and content field = Lifestyle / Disease Management. *P* values for the odds ratios are calculated in comparison with this reference category.

## Abbreviations

AI: artificial intelligence
CLT: cognitive load theory
LLM: large language model
OR: odds ratio
SP: standardized patient
STROBE: Strengthening the Reporting of Observational Studies in Epidemiology
