# Supplementary material for: Impact of GPT-4–Generated Discharge Letters on Patients’ Medical Comprehension: Prospective Crossover Study
Source: J Med Internet Res. 2026 Feb 26;28:e81243. doi: 10.2196/81243 (PMC12982961; doi:10.2196/81243)
Supplement: Multimedia Appendix 1 [file jmir_v28i1e81243_app1.pdf]

GPT- basierter Patientenzentrierter Brief
Arterielle Hypertonie
Ambulante Vorstellung

Liebe/r R. Raiser,

Zeit und Ort der Behandlung

Die ärztliche Vorstellung von R. Raiser war in einer nephrologischen Ambulanz zur Mitbeurteilung und Abklärung des hohen Blutdrucks. Dieser Besuch war also ambulant, das heißt, R. Raiser musste nicht im Krankenhaus bleiben, sondern war nur für die Untersuchung dort.

Hauptdiagnose

Die Hauptdiagnose ist ein arterieller Bluthochdruck, auch Hypertonie genannt, der Stufe II. Das bedeutet, dass der Blutdruck von Herrn Raiser öfter viel zu hoch ist. Das kann auf Dauer zu Schäden an den Organen führen, zum Beispiel an den Nieren.

Nebendiagnosen

Zustand nach Entzündung der Speiseröhre wegen Rückflusses von Magensäure.
Zustand nach einer Kniegelenkspiegelung am rechten Knie.
Eine zu hohe Menge an Cholesterin im Blut, was Hypercholesterinämie genannt wird.
Vermeiden Sie die Einnahme von Codein und Azithromycin wegen Ihrer Allergie.
Diese sind Wirkstoffe, die in bestimmten Medikamenten vorkommen.

Was ist im Krankenhaus passiert?

Während Ihres Krankenhausaufenthalts wurden verschiedene Untersuchungen durchgeführt, um genauer zu verstehen, warum Ihr Blutdruck erhöht ist und um andere mögliche gesundheitliche Probleme auszuschließen. Sie haben einen Bluthochdruck, der medizinisch als "arterielle Hypertonie" bezeichnet wird. Die Ärzte wollten herausfinden, ob es eine spezielle Ursache dafür gibt, oder ob es sich um eine

sogenannte "essentielle" Hypertonie handelt, was bedeutet, dass keine spezifische Ursache gefunden werden kann.

Hier ist eine Liste der Dinge, die gemacht wurden:

1. Es wurden bildgebende Untersuchungen von Ihren Nieren und Nebennieren mittels Ultraschall durchgeführt. Dabei hat man alles als normal befunden.
2. Es gab eine MRT-Untersuchung im August, um zu sehen, ob Ihre Nierenarterien verengt sind, was Ihren Blutdruck erhöhen könnte. Die Untersuchung hat keine Probleme gezeigt.
3. Man hat Ihre Blut- und Urinwerte untersucht, um andere Krankheiten auszuschließen, die hohen Blutdruck verursachen könnten, wie z.B. einen erhöhten Cortisolspiegel (ein Stresshormon) oder Störungen im Hormonhaushalt. Das alles war unauffällig.
4. Ihre Nierenfunktion wurde überprüft und ist normal.
5. Es wurde festgestellt, dass Sie eine leicht erhöhte Eiweißmenge im Urin haben (Proteinurie), was ein Zeichen für Nierenschäden sein kann, die durch hohen Blutdruck verursacht werden.
6. Sie hatten eine Reflux-Krankheit, bei der Magensäure in die Speiseröhre zurückfließt, und eine Arthroskopie (ein gelenkschonendes Operationsverfahren) am Knie.
7. Außerdem haben Sie einen höheren Cholesterinspiegel und Allergien gegen bestimmte Medikamente.

Die Ärzte haben Ihre Medikamente neu eingestellt, um Ihren Blutdruck besser zu kontrollieren. Sie nehmen zukünftig eine Kombination aus drei Wirkstoffen (Ramipril, Amlodipin und HCT) einmal täglich ein. Die vorherige Medikation mit Ramipril allein und Amlodipin allein wurde gestoppt.

Zur weiteren Kontrolle wurden Sie gebeten, eine 24-Stunden-Urinprobe zu Hause zu sammeln, um nach Stoffwechselprodukten zu suchen, die auf einen seltenen Tumor hinweisen könnten, der den Blutdruck beeinflusst. Ein Behälter dafür wurde Ihnen mitgegeben.

Die Ärzte empfehlen Ihnen, zu Hause regelmäßig Ihren Blutdruck zu messen und aufzuschreiben, um sicherzustellen, dass die neuen Medikamente wirken. Wenn der Blutdruck sehr hoch steigt (über 180/120 mmHg), sollten Sie sofort einen Arzt aufsuchen, um Schäden an wichtigen Organen zu vermeiden.

Sie sollten auch versuchen, Ihr Gewicht zu reduzieren, weniger Salz zu essen und eine gesunde Ernährung mit viel Obst und Gemüse sowie fettarmen Milchprodukten zu befolgen. Es wird empfohlen, einen Body-Mass-Index (BMI) zwischen 18,5 und 24,9 zu erreichen.

Insgesamt deuten Ihre Untersuchungsergebnisse darauf hin, dass es keine spezifische Ursache für Ihren hohen Blutdruck gibt, und die Ärzte gehen von einer "essentiellen Hypertonie" aus, die mit Medikamenten und Lebensstiländerungen kontrolliert werden sollte.

Was müssen Sie jetzt beachten?

Medikamente:
Das neue Medikament ist eine Kombipille, die aus drei Wirkstoffen besteht, um den Blutdruck zu senken. Sie nehmen davon täglich morgens eine Tablette. Die Zahlen geben an, wie stark jede Komponente dosiert ist: 10 mg für den Teil, der die Blutgefäße entspannt, 10 mg für den Teil, der die Blutgefäßwände geschmeidig macht, und 25 mg für den Bestandteil, der die Wasserausscheidung erhöht. Die beiden anderen Medikamente, die jeweils nur einen Wirkstoff enthielten, sollen nicht mehr genommen werden. Das Mittel für den Magen bleibt unverändert bei einer Tablette täglich morgens.

Ramipril/Amlodipin/HCT	10/10/25 mg	1 – 0 – 0	NEU
Ramipril	5 mg	1 – 0 – 1	BEENDET
Amlodipin	10 mg	1 – 0 – 0	BEENDET
Pantoprazol	20 mg	1 – 0 – 0	

Ernährung / Bewegung:
Ihr Blutdruck sollte im Allgemeinen unter 130/80 bleiben. Sie sollten regelmäßig Ihren Blutdruck überprüfen und die Messergebnisse festhalten. Falls die Werte einmal deutlich höher sein sollten, gehen Sie sofort zum Arzt. Ihre Ernährung sollte viel Obst und Gemüse sowie Milchprodukte mit wenig Fett beinhalten. Achten Sie darauf, nicht mehr als 5 bis 6 Gramm Salz pro Tag zu sich zu nehmen. Halten Sie Ihr Körpergewicht in einem Bereich, der als normal gilt, gemessen am Körper-Masse-Index (BMI). Vermeiden Sie Grapefruitsaft, weil er die Wirkung Ihrer Medikamente stören kann.

Weitere Arztkontakte / Untersuchungen:
Einer Ihrer anstehenden Tests beinhaltet das Sammeln von Urin über einen ganzen Tag. Dieser Test hilft herauszufinden, ob Sie eine bestimmte seltene Erkrankung haben, die den Blutdruck stark erhöhen kann. Außerdem sollten Sie untersuchen lassen, ob Sie an einer Schlafstörung leiden, bei der die Atmung während des Schlafs immer wieder kurze Zeit aussetzt bei der der Blutdruck steigen kann.

Bitte besprechen Sie Ihren neuen Medikamentenplan unbedingt mit Ihrem Hausarzt oder Ihrer Hausärztin.

GPT-based patient-centered letter
Arterial hypertension
Outpatient consultation

Dear R. Raiser,

Time and place of treatment

R. Raiser's medical consultation took place in a nephrology outpatient clinic for the co-assessment and clarification of high blood pressure. This visit was therefore outpatient, meaning that R. Raiser did not have to stay in the hospital, but was only there for the examination.

Primary diagnosis

The primary diagnosis is stage II arterial hypertension, also known as high blood pressure. This means that Mr. Raiser's blood pressure is often much too high. Over time, this can lead to damage to organs, such as the kidneys.

Secondary diagnoses

Condition following inflammation of the esophagus due to reflux of stomach acid.

Condition following arthroscopy of the right knee.

Excessive amount of cholesterol in the blood, known as hypercholesterolemia.

Avoid taking codeine and azithromycin due to your allergy. These are active ingredients found in certain medications.

What happened at the hospital?

During your hospital stay, various tests were performed to better understand why your blood pressure is elevated and to rule out other possible health problems. You have high blood pressure, which is medically referred to as "arterial hypertension." The doctors wanted to find out if there was a specific cause for this or if it was a case of so-called "essential" hypertension, which means that no specific cause can be found.

Here is a list of the things that were done:

1. Imaging tests were performed on your kidneys and adrenal glands using ultrasound. Everything was found to be normal.

2. An MRI scan was performed in August to see if your renal arteries were narrowed, which could increase your blood pressure. The scan did not reveal any problems.

3. Your blood and urine values were examined to rule out other diseases that could cause high blood pressure, such as elevated cortisol levels (a stress hormone) or hormonal imbalances. All of these were normal.

4. Your kidney function was checked and is normal.

5. It was found that you have a slightly elevated amount of protein in your urine (proteinuria), which can be a sign of kidney damage caused by high blood pressure.

6. You had reflux disease, in which stomach acid flows back into the esophagus, and arthroscopy (a joint-friendly surgical procedure) on your knee.

7. You also have high cholesterol and allergies to certain medications.

The doctors have adjusted your medication to better control your blood pressure. In the future, you will take a combination of three active ingredients (ramipril, amlodipine, and HCT) once a day. The previous medication with ramipril alone and amlodipine alone has been discontinued.

For further monitoring, you have been asked to collect a 24-hour urine sample at home to check for metabolites that could indicate a rare tumor that affects blood pressure. You have been given a container for this purpose.

The doctors recommend that you regularly measure and record your blood pressure at home to ensure that the new medication is working. If your blood pressure rises very high (above 180/120 mmHg), you should see a doctor immediately to prevent damage to vital organs.

You should also try to lose weight, eat less salt, and follow a healthy diet with plenty of fruit and vegetables and low-fat dairy products. It is recommended that you achieve a body mass index (BMI) between 18.5 and 24.9.

Overall, your test results indicate that there is no specific cause for your high blood pressure, and doctors assume that you have “essential hypertension,” which should be controlled with medication and lifestyle changes.

What do you need to keep in mind now?

Medication:

The new medication is a combination pill that consists of three active ingredients to lower blood pressure. You take one tablet daily in the morning. The numbers indicate the dosage of each component: 10 mg for the part that relaxes the blood vessels, 10 mg for the part that makes the blood vessel walls more flexible, and 25 mg for the component that increases water excretion. The other two medications, each of which contained only one active ingredient, should no longer be taken. The medication for the stomach remains unchanged at one tablet daily in the morning.

Ramipril/Amlodipine/HCT 10/10/25 mg	1 – 0 – 0	NEW
Ramipril 5 mg	1 – 0 – 1	DISCONTINUED
Amlodipine 10 mg	1 – 0 – 0	DISCONTINUED
Pantoprazole 20 mg	1 – 0 – 0	

Diet/exercise:

Your blood pressure should generally remain below 130/80. You should check your blood pressure regularly and record the results. If the values are significantly higher, see your doctor immediately. Your diet should include plenty of fruit and vegetables as well as low-fat dairy products. Make sure you do not consume more than 5 to 6 grams of salt per day. Maintain your body weight within a range that is considered normal, as measured by your body mass index (BMI). Avoid grapefruit juice, as it can interfere with the effectiveness of your medication.

Further doctor's appointments / tests:

One of your upcoming tests involves collecting urine over a whole day. This test will help determine whether you have a rare condition that can cause a sharp rise in blood pressure. You should also be tested for a sleep disorder in which your breathing repeatedly stops for short periods during sleep, which can cause your blood pressure to rise.

Be sure to discuss your new medication plan with your family doctor.

GPT- basierter Patientenzentrierter Brief
Erstdiagnose Diabetes mellitus
Stationärer Aufenthalt

Liebe/r S. Süß,

Zeit und Ort der Behandlung

Die ärztliche Vorstellung war im Oktober 2023. Sie fand in einer Klinik statt und es war ein stationärer Aufenthalt. Das bedeutet, dass Sie dort übernachtet haben.

Hauptdiagnose

Sie haben zum ersten Mal erfahren, dass Sie Zuckerkrankheit (Diabetes Typ 2) haben. Ihr Blutzucker war sehr hoch, was zu einem ungesunden Gewichtsverlust und häufigem Wasserlassen geführt hat.

Nebendiagnosen

Sie haben Verschleiß in der Hüfte (Coxarthrose) auf der linken Seite.
Sie hatten in der Vergangenheit (1972) eine Blinddarm-Operation.
Sie hatten eine Nagelpilzinfektion an beiden Füßen.
Sie sind allergisch gegen Pollen (Heuschnupfen).

Was ist im Krankenhaus passiert?

In verständlicher Sprache kann man Ihren Krankenhausaufenthalt folgendermaßen zusammenfassen:

Sie wurden ins Krankenhaus eingewiesen, weil Ihr Blutzucker sehr hoch war – das war das erste Anzeichen dafür, dass Sie Typ-2-Diabetes haben. Man hat festgestellt, dass Sie in letzter Zeit ungewollt Gewicht verloren haben und übermäßig viel uriniert haben. Ein Bluttest hat gezeigt, dass Ihr Langzeitblutzuckerwert (HbA1c) bei 14,1% liegt, was deutlich über dem Normalwert ist. Die Ärzte haben getestet, ob Ihr Körper noch ausreichend eigenes Insulin produziert, was der Fall ist, und ob es Anzeichen für einen speziellen Diabetes-Typ gibt, bei dem das Immunsystem die Insulinproduzierenden Zellen angreift – das ist nicht der Fall. Mit einer Ultraschalluntersuchung hat man geschaut, ob es

Auffälligkeiten an Ihrer Bauchspeicheldrüse gibt – es wurde nichts Ungewöhnliches gefunden.

Während Ihres Aufenthaltes, hat man damit begonnen, Ihren hohen Blutzucker zu behandeln, indem man Ihnen Flüssigkeit gegeben hat und mit Insulin, einem Medikament, welches normalerweise von der Bauchspeicheldrüse produziert wird, um Ihren Blutzucker zu senken. Dazu haben Sie auch noch zwei weitere Medikamente bekommen, Metformin und Empagliflozin, die dabei helfen, den Blutzucker zu kontrollieren.

Weil Ihr Blutzucker vorher nicht behandelt wurde, haben Sie schon eine Nervenschädigung in den Füßen, das nennt man Polyneuropathie. Man hat festgestellt, dass das Empfinden in Ihren Füßen herabgesetzt ist. Ihre Nieren funktionieren zum Glück normal, was man bei Diabetes auch immer prüfen muss.

Außerdem haben die Ärzte Ihr Cholesterin untersucht und festgestellt, dass es auch zu hoch ist. Deshalb sollten Sie jetzt ein Medikament namens Atorvastatin einnehmen, um den Cholesterinspiegel zu senken.

Sie haben auch Ratschläge bekommen, wie Sie Ihren Blutzucker zu Hause kontrollieren können. Wenn Ihr Blutzucker am Morgen vor dem Frühstück an drei aufeinander folgenden Tagen zu hoch ist, sollen Sie etwas mehr Insulin nehmen. Wenn Ihr Blutzucker vor dem Frühstück zu niedrig ist, sollen Sie weniger Insulin nehmen. Ihr Arzt hat Ihnen empfohlen, regelmäßig zu verschiedenen Fachärzten (Augenarzt, Herzspezialist und Nierenspezialist) zu gehen, um sicherzustellen, dass der Diabetes keine weiteren Probleme verursacht.

Es ist wichtig, dass Sie regelmäßig Ihre Füße selbst untersuchen, um Probleme früh zu erkennen, da die Nervenschädigung in Ihren Füßen das Risiko für weitere Fußprobleme erhöht. Zusätzlich hat man Ihnen geraten, auf Ihre Ernährung zu achten und regelmäßig aktiv zu sein, um Ihr Herz gesund zu halten. Außerdem sollten Sie Ihren regelmäßigen Alkoholkonsum reduzieren, da dieser den Blutzuckerspiegel beeinflussen kann.

Am Ende Ihres Krankenhausaufenthalts ging es Ihnen besser, und Sie wurden mit der Anweisung entlassen, dass Sie den neuen Plan zur Kontrolle Ihres Diabetes befolgen sollen.

Was müssen Sie jetzt beachten?

Medikamente

- Metformin: Nehmen Sie täglich zwei Tabletten zu je 500 mg, eine am Morgen und eine am Abend.
- Empagliflozin: Nehmen Sie täglich 10 mg am Morgen ein.
- Insulin glargin: Spritzen Sie täglich 14 Einheiten um 8 Uhr morgens.
    - Falls Ihr morgendlicher Blutzuckerwert über 150 liegt, spritzen Sie 2 Einheiten mehr Insulin.
    - Falls Ihr morgendlicher Blutzuckerwert unter 100 liegt, nehmen Sie 4 Einheiten weniger.
- Atorvastatin: Jeden Morgen eine Tablette mit 20 mg einnehmen.

<u>Ihr vollständiger Medikamentenplan:</u>

Metformin 500 mg	1-0-1	NEU
Empagliflozin 10 mg	1-0-0	NEU
Insulin glargin 100 E/ml	14 E um 8 Uhr morgens	NEU
Atorvastatin 20 mg	1-0-0	NEU

<u>Ernährung / Bewegung</u>

- Beschwerden: Wenn man sich schwach fühlt oder zittert, könnte dies ein Zeichen für zu niedrigen Blutzucker sein.
- Versuchen Sie, weniger Alkohol zu trinken oder ganz darauf zu verzichten, denn Alkohol kann Ihren Blutzuckerspiegel beeinflussen.
- Körperliche Aktivität: Fügen Sie Ihrer Wochenroutine Übungen hinzu, die Sie leicht ins Schwitzen bringen (wie zügiges Gehen), das sollten 150 Minuten pro Woche sein.

<u>weitere Arztkontakte / Untersuchungen</u>

- Kontrollen beim Augenarzt: Lassen Sie Ihre Augen regelmäßig überprüfen, um sicherzustellen, dass Ihre Sehkraft nicht durch den Diabetes beeinträchtigt wird.
- Untersuchungen beim Herz-Spezialisten: Besuche beim Herz-Spezialisten sind wichtig, um Ihr Herz gesund zu halten.
- Untersuchungen beim Nieren-Spezialisten: Besuche beim Nieren-Spezialisten sind wichtig, um sicherzugehen, dass Ihre Nieren gut arbeiten.
- Langzeitblutzuckerwert (HbA1c-Wert): Dieser Wert sollte alle drei Monate überprüft werden, er zeigt an, wie gut Ihr Blutzuckerspiegel über einen längeren Zeitraum kontrolliert wurde. Das Ziel ist es, den Wert ohne starke Schwankungen im Blutzuckerlevel in einem bestimmten Bereich (zwischen 7,5 und 8 %) zu halten.
- Fußinspektionen: Lassen Sie regelmäßig Ihre Füße von einem Fachmann überprüfen, um frühzeitig zu merken, wenn Sie Probleme wegen Ihres Diabetes bekommen.

Bitte besprechen Sie Ihren neuen Medikamentenplan unbedingt mit Ihrem Hausarzt oder Ihrer Hausärztin.

GPT-based patient-centered letter

Initial diagnosis of diabetes mellitus Inpatient stay

Dear S. Süß,

Time and place of treatment

The medical consultation took place in October 2023. It took place in a clinic and was an inpatient stay. This means that you stayed overnight there.

Primary diagnosis

You were diagnosed with diabetes (type 2) for the first time. Your blood sugar was very high, which led to unhealthy weight loss and frequent urination.

Secondary diagnoses

You have wear and tear in your left hip (coxarthrosis).

You had an appendectomy in the past (1972).

You had a fungal nail infection on both feet.

You are allergic to pollen (hay fever).

What happened at the hospital?

In plain language, your hospital stay can be summarized as follows:

You were admitted to the hospital because your blood sugar was very high—this was the first sign that you have type 2 diabetes. It was found that you had recently lost weight unintentionally and were urinating excessively. A blood test showed that your long-term blood sugar level (HbA1c) is 14.1%, which is significantly above normal. The doctors tested whether your body still produces enough insulin, which it does, and whether there are signs of a specific type of diabetes in which the immune system attacks the insulin-producing cells – this is not the case. An ultrasound examination was performed to check for abnormalities in your pancreas – nothing unusual was found.

During your stay, they began treating your high blood sugar by giving you fluids and insulin, a medication normally produced by the pancreas, to lower your blood sugar. You were also given two other medications, metformin and empagliflozin, which help control blood sugar.

Because your blood sugar was not treated previously, you already have nerve damage in your feet, which is called polyneuropathy. It has been determined that the sensation in your feet is reduced. Fortunately, your kidneys are functioning normally, which is always necessary to check in cases of diabetes.

The doctors also tested your cholesterol and found that it is too high. You should now take a medication called atorvastatin to lower your cholesterol level.

You have also been given advice on how to control your blood sugar at home. If your blood sugar is too high in the morning before breakfast on three consecutive days, you should take a little more insulin. If your blood sugar is too low before breakfast, you should take less insulin. Your doctor has recommended that you see various specialists (ophthalmologist, cardiologist, and nephrologist) regularly to ensure that your diabetes does not cause any further problems.

It is important that you check your feet regularly yourself to detect problems early, as nerve damage in your feet increases the risk of further foot problems. In addition, you have been advised to watch your diet and be physically active on a regular basis to keep your heart healthy. You should also reduce your regular alcohol consumption, as this can affect your blood sugar levels.

At the end of your hospital stay, you were feeling better and were discharged with instructions to follow the new plan for managing your diabetes.

What do you need to keep in mind now?

<u>Medications</u>

- Metformin: Take two 500 mg tablets daily, one in the morning and one in the evening.
- Empagliflozin: Take 10 mg daily in the morning.
- Insulin glargine: Inject 14 units daily at 8 a.m.
    - If your morning blood sugar level is above 150, inject 2 more units of insulin.
    - If your morning blood sugar level is below 100, take 4 fewer units.
- Atorvastatin: Take one 20 mg tablet every morning.

<u>Your complete medication schedule:</u>

Metformin 500 mg	1-0-1	NEW
Empagliflozin 10 mg	1-0-0	NEW
Insulin glargine 100 E/ml	14 E at 8 a.m.	NEW
Atorvastatin 20 mg	1-0-0	NEW

Diet / Exercise

- Symptoms: If you feel weak or shaky, this could be a sign of low blood sugar.
- Try to drink less alcohol or avoid it altogether, as alcohol can affect your blood sugar levels.
- Physical activity: Add exercises that make you sweat a little (such as brisk walking) to your weekly routine; this should amount to 150 minutes per week.

Further doctor's appointments / examinations

- Checkups with an ophthalmologist: Have your eyes checked regularly to ensure that your vision is not impaired by diabetes.
- Checkups with a cardiologist: Visits to a cardiologist are important for keeping your heart healthy.
- Checkups with a nephrologist: Visits to a nephrologist are important to ensure that your kidneys are working properly.
- Long-term blood sugar level (HbA1c value): This value should be checked every three months; it indicates how well your blood sugar level has been controlled over a longer period of time. The goal is to keep the value within a certain range (between 7.5 and 8%) without significant fluctuations in blood sugar levels.
- Foot inspections: Have your feet checked regularly by a specialist to detect any problems caused by your diabetes at an early stage.

Be sure to discuss your new medication plan with your family doctor.

GPT- basierter Patientenzentrierter Brief
Diabetische Nephropathie
Stationärer Aufenthalt

Liebe/r M. Maurer,

Zeit und Ort der Behandlung

Die Untersuchung war in einer Klinik und es handelte sich um einen stationären Aufenthalt, da Sie dort mehrere Tage waren und unter anderem eine Nierenbiopsie (also eine Gewebeprobe der Niere) erhalten haben.

Hauptdiagnose

Sie haben eine chronische Nierenkrankheit (das bedeutet, Ihre Nieren arbeiten nicht mehr richtig und das schon seit längerer Zeit). Diese Nierenkrankheit ist durch Diabetes (Zuckerkrankheit) und Bluthochdruck bedingt und befindet sich in einem mittleren Stadium (G3b), was anzeigt, dass die Nierenfunktion schon etwas ernster beeinträchtigt ist.

Nebendiagnosen

Diabetes mellitus Typ 2 seit dem Jahr 2017: Das ist die Zuckerkrankheit, bei der der Blutzuckerspiegel zu hoch ist. Aktuelle Blutzuckerwerte waren zuletzt nicht im optimalen Bereich.
Adipositas II°: Das bedeutet, Sie sind stark übergewichtig mit einem Body-Mass-Index (BMI) von 35,62 kg/m^2, was bedeutet, dass das Körpergewicht zu Ihrer Körpergröße im Vergleich zu hoch ist.
Arterielle Hypertonie: Das ist der medizinische Begriff für Bluthochdruck, den Sie ebenfalls seit 2017 haben.
Zustand nach Kniegelenksersatz (TEP) auf der rechten Seite im Mai 2019 wegen starker Abnutzung des Gelenks nach einem Skiunfall: TEP ist die Abkürzung für eine künstliches Kniegelenk.

Was ist im Krankenhaus passiert?

Während Ihres Krankenhausaufenthalts stand die Untersuchung Ihrer Nieren im Vordergrund. Sie haben eine chronische Nierenkrankheit, die durch Diabetes und hohen Blutdruck verursacht wurde. Weil Ihre Nieren schlechter arbeiteten und mehr Eiweiß im Urin gefunden wurde als normal, wurde eine Nierenbiopsie gemacht. Das ist eine

Untersuchung, bei der eine kleine Probe des Nierengewebes entnommen und unter dem Mikroskop analysiert wird.

Die Ergebnisse der Biopsie zeigen, dass Ihre Nieren aufgrund der Schäden durch den Diabetes und den hohen Blutdruck verändert sind. Etwa 40% Ihrer Nierenstruktur sind bereits dauerhaft geschädigt (das heißt durch Narbengewebe ersetzt). Dies bestätigt die Diagnose der diabetischen und durch hohen Blutdruck verursachten Nierenerkrankung.

Außerdem wurde Ihr Blutzucker kontrolliert und festgestellt, dass dieser trotz der Medikamente, die Sie nehmen (Metformin), zu hoch ist. Deshalb haben die Ärzte Ihre Behandlung angepasst und ein neues Medikament hinzugefügt, das helfen soll, den Blutzucker zu senken und die Nierenerkrankung nicht schneller voranschreiten zu lassen.

Es wurde auch festgestellt, dass Sie zu hohen Blutdruck haben, und dieser wurde mit weiteren Medikamenten behandelt. Zusätzlich zu den Medikamenten haben Sie ein Medikament erhalten, das überschüssiges Wasser aus Ihrem Körper entfernt (Diuretikum), weil Sie Wasseransammlungen in den Beinen hatten.

Es ist wichtig, dass Sie auch nach Ihrer Entlassung aus dem Krankenhaus weiterhin regelmäßige ärztliche Kontrollen haben. Die Ärzte empfehlen, zunächst alle drei Monate Ihre Nieren von einem Nierenfacharzt (Nephrologe) überprüfen zu lassen.

Wichtig für Sie ist auch, dass Sie Ihr Gewicht reduzieren und sich gesund ernähren, um Ihre Nieren zu schützen und Ihren Diabetes besser in den Griff zu bekommen. Außerdem sollten Sie auf Medikamente, die Ihre Nieren schädigen können, verzichten, wie das Schmerzmittel Ibuprofen, das Sie aufgrund von Knieschmerzen genommen haben.

Falls Sie Fieber oder eine Infektion bekommen, sollten Sie zwei bestimmte Diabetesmedikamente (Metformin und Forxiga) vorübergehend absetzen, um schwere Nebenwirkungen zu vermeiden. Und weil Sie eine Penicillinallergie haben, sollte das bei der Behandlung von Infektionen beachtet werden.

Alles in allem war Ihr Zustand bei der Entlassung stabil, und es gab keine Komplikationen nach der Nierenbiopsie. Nun ist es wichtig, dass Sie die Ratschläge und Anpassungen in der Medikation befolgen und regelmäßig zu den empfohlenen Kontrolluntersuchungen gehen.

Was müssen Sie jetzt beachten?

Medikamente:

- Ramipril/Amlodipin: Diese sind Blutdrucksenker. Die Menge, die Sie nehmen sollten, wurde von einer auf zwei Tabletten am Morgen erhöht.
- Metformin: Das ist ein Medikament für Menschen mit Zuckerkrankheit (Diabetes). Sie sollten jetzt morgens und abends jeweils nur eine Tablette nehmen, also weniger als vorher.
- Forxiga: Das ist auch ein Medikament für Diabetes. Sie sollen jeden Morgen eine Tablette davon nehmen.

- Torasemid: Das ist ein Medikament, das den Körper entwässert, also dafür sorgt, dass Sie mehr pinkeln und so überschüssiges Wasser loswerden. Sobald sich Ihre Flüssigkeitsmengen eingependelt haben, soll die Menge verringert werden.
- Ibuprofen: Das ist ein Schmerzmittel, das Sie aber bitte nicht nehmen sollen, weil es schlecht für Ihre Nieren sein könnte.

<u>Ihr vollständiger Medikamentenplan:</u>

Ramipril/Amlodipin 5 mg/5 mg	2 – 0 – 0	GESTEIGERT, war vorher 1 – 0 – 0
HCT 12,5 mg	1 – 0 – 0	
Metformin 500 mg	1 – 0 – 1	REDUZIERT, war vorher 2 – 0 – 2
Forxiga 10 mg	1 – 0 – 0	NEU
Torasemid 20 mg	1 – 0 – 0	im Verlauf reduzieren
Atorvastatin 40mg	0 – 0 – 1	
Vigantoletten 1000IE	1 – 0 – 0	

<u>Ernährung / Bewegung</u>

- Gewichtsmanagement: Es könnte gut sein, wenn Sie sich professionelle Hilfe suchen, um Gewicht zu verlieren.
- Diät: Probieren Sie die mediterrane Diät, die viel Gemüse, Früchte, Nüsse, Samen, Olivenöl und Fisch beinhaltet. Diese Ernährung kann helfen, Ihre Nieren und Ihren Blutzuckerspiegel gesund zu halten und beim Abnehmen helfen.

<u>weitere Arztkontakte / Untersuchungen</u>

- Nierenfunktion: Alle drei Monate sollten Sie Ihre Nieren von einem Nierenspezialisten (Nephrologen) untersuchen lassen, um sicherzustellen, dass sie gut arbeiten.
- Blutdruck: Messen Sie regelmäßig Ihren Blutdruck zu Hause und stimmen Sie Änderungen an Ihren Medikamenten mit Ihrem Arzt ab.
- Penicillinallergie: Weil Sie allergisch auf Penicillin sind, sollten Sie keine Medikamente nehmen, die Penicillin enthalten, da dies zu einer allergischen Reaktion führen könnte. Teilen Sie dies jedem Arzt mit.
- GLP1 Analoga: Diese Gruppe von Medikamenten wird normalerweise für Diabetes Typ 2 verwendet, Sie sollten sie aber meiden, weil sie Magen-Darm-Probleme als Nebenwirkungen haben können.

Bitte besprechen Sie Ihren neuen Medikamentenplan unbedingt mit Ihrem Hausarzt oder Ihrer Hausärztin.

GPT-based patient-centered letter Diabetic Kidney Disease (DKD) Inpatient stay

Dear M. Maurer,

Time and place of treatment

The examination took place in a clinic and involved an inpatient stay, as you were there for several days and underwent a kidney biopsy (i.e., a tissue sample was taken from your kidney), among other things.

Primary diagnosis

You have chronic kidney disease (this means that your kidneys have not been functioning properly for some time). This kidney disease is caused by diabetes and high blood pressure and is at a moderate stage (G3b), which indicates that your kidney function is already somewhat severely impaired.

Secondary diagnoses

Type 2 diabetes mellitus since 2017: This is diabetes in which blood sugar levels are too high. Current blood sugar levels were not within the optimal range.
Obesity II°: This means you are severely overweight with a body mass index (BMI) of 35.62 kg/m^2, which means that your body weight is too high in relation to your height.
Arterial hypertension: This is the medical term for high blood pressure, which you have also had since 2017.
Condition after knee replacement (TEP) on the right side in May 2019 due to severe wear and tear of the joint after a skiing accident: TEP is the abbreviation for an artificial knee joint.

What happened in the hospital?

During your hospital stay, the focus was on examining your kidneys. You have chronic kidney disease caused by diabetes and high blood pressure. Because your kidneys were not working as well as they should and more protein than normal was found in your urine, a kidney biopsy was performed. This is a test in which a small sample of kidney tissue is taken and analyzed under a microscope.

The results of the biopsy show that your kidneys have been altered due to damage caused by diabetes and high blood pressure. Approximately 40% of your kidney structure has already been permanently damaged (i.e., replaced by scar tissue). This confirms the diagnosis of diabetic kidney disease caused by high blood pressure.
In addition, your blood sugar was checked and found to be too high despite the medication you are taking (metformin). Therefore, the doctors have adjusted your treatment and added a new medication to help lower your blood sugar and prevent your kidney disease from progressing more rapidly.
It was also found that you have high blood pressure, which was treated with additional medication. In addition to the medication, you were given a drug that removes excess water from your body (diuretic) because you had water retention in your legs.
It is important that you continue to have regular medical check-ups after you are discharged from the hospital. The doctors recommend that you have your kidneys checked by a kidney specialist (nephrologist) every three months at first.
It is also important for you to lose weight and eat a healthy diet to protect your kidneys and better control your diabetes. You should also avoid medications that can damage your kidneys, such as the painkiller ibuprofen, which you took for knee pain.
If you develop a fever or infection, you should temporarily stop taking two specific diabetes medications (metformin and Forxiga) to avoid serious side effects. And because you are allergic to penicillin, this should be taken into account when treating infections.
All in all, your condition was stable at discharge and there were no complications after the kidney biopsy. Now it is important that you follow the advice and adjustments to your medication and attend the recommended check-ups regularly.

What do you need to keep in mind now?

<u>Medications:</u>

- Ramipril/amlodipine: These are blood pressure medications. The amount you should take has been increased from one to two tablets in the morning.
- Metformin: This is a medication for people with diabetes. You should now take only one tablet in the morning and one in the evening, which is less than before.
- Forxiga: This is also a medication for diabetes. You should take one tablet every morning.
- Torasemide: This is a medication that dehydrates the body, i.e., it makes you urinate more and thus get rid of excess water. Once your fluid levels have stabilized, the amount should be reduced.
- Ibuprofen: This is a painkiller, but please do not take it as it could be bad for your kidneys.

<u>Your complete medication plan:</u>

Ramipril/Amlodipine 5 mg/5 mg	2 – 0 – 0	INCREASED, was previously 1 – 0 – 0

HCT 12.5 mg	1 – 0 – 0	
Metformin 500 mg	1 – 0 – 1	REDUCED, was previously 2 – 0 – 2
Forxiga 10 mg	1 – 0 – 0	NEW
Torasemide 20 mg	1 – 0 – 0	Reduce over time
Atorvastatin 40mg	0 – 0 – 1	
Vigantoletten 1000IE	1 – 0 – 0	

Diet / Exercise

- Weight management: It may be a good idea to seek professional help to lose weight.
- Diet: Try the Mediterranean diet, which includes lots of vegetables, fruits, nuts, seeds, olive oil, and fish. This diet can help keep your kidneys and blood sugar levels healthy and help you lose weight.
-

Further doctor's appointments / examinations

- Kidney function: Every three months, you should have your kidneys checked by a kidney specialist (nephrologist) to make sure they are working well.
- Blood pressure: Measure your blood pressure regularly at home and discuss any changes to your medication with your doctor.
- Penicillin allergy: Because you are allergic to penicillin, you should not take any medications that contain penicillin, as this could cause an allergic reaction. Tell every doctor about this.
- GLP1 analogues: This group of medications is usually used for type 2 diabetes, but you should avoid them because they can cause gastrointestinal problems as side effects.

Be sure to discuss your new medication plan with your family doctor.
