# Supplementary figures and images for: Impact of GPT-4–Generated Discharge Letters on Patients’ Medical Comprehension: Prospective Crossover Study
Source: J Med Internet Res. 2026 Feb 26;28:e81243. doi: 10.2196/81243 (PMC12982961; doi:10.2196/81243)

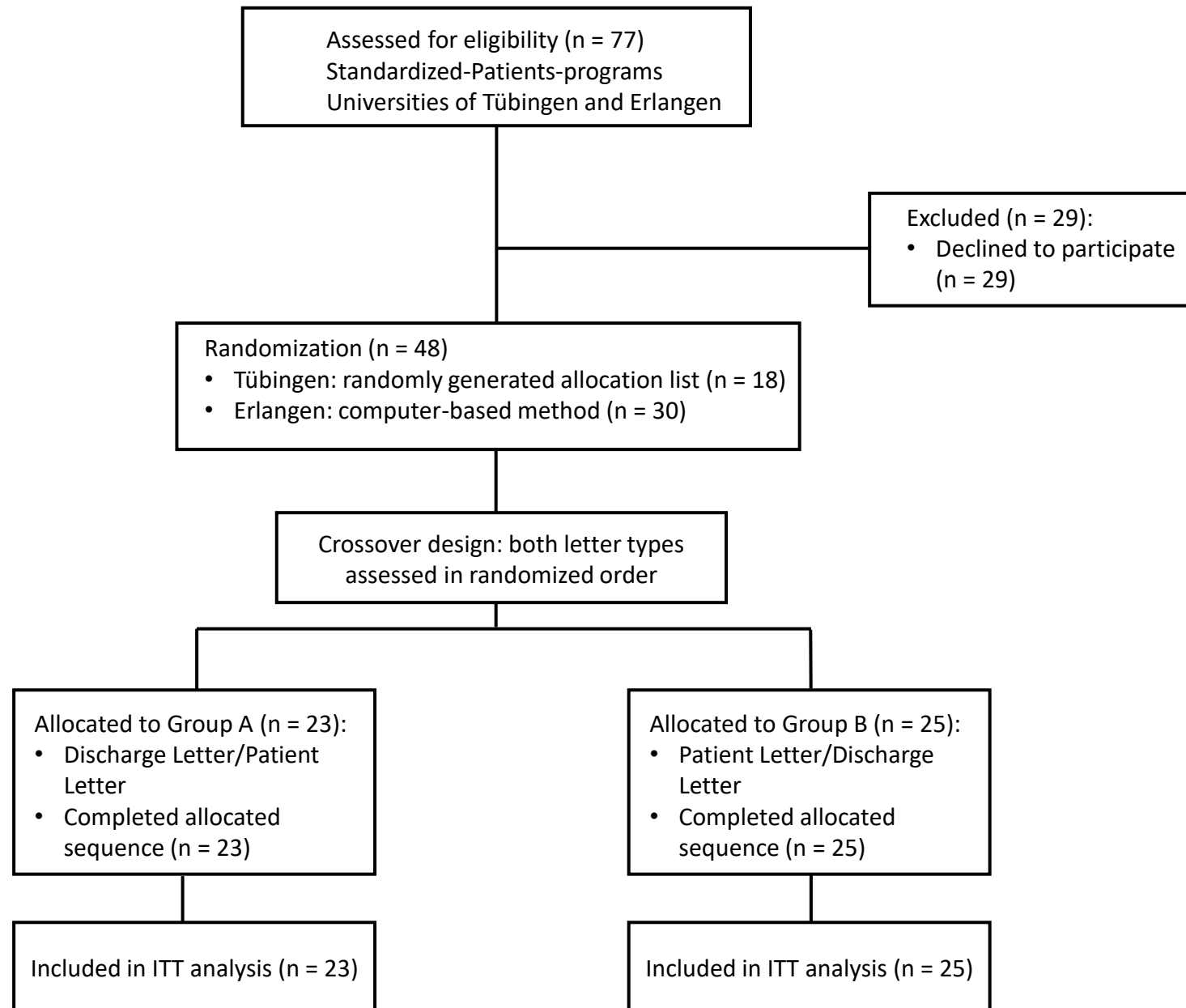

Supplement: Multimedia Appendix 2 [file jmir_v28i1e81243_app2.pdf]

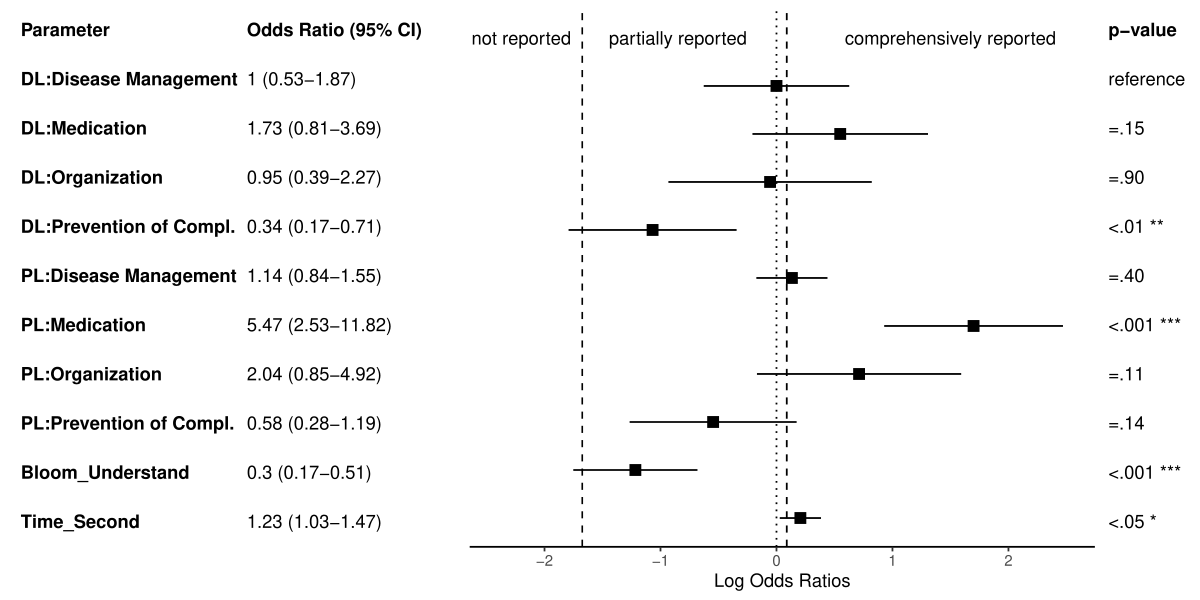

Supplement: Multimedia Appendix 9 [file jmir_v28i1e81243_app9.png]
