# Supplementary material for: Impact of GPT-4–Generated Discharge Letters on Patients’ Medical Comprehension: Prospective Crossover Study
Source: J Med Internet Res. 2026 Feb 26;28:e81243. doi: 10.2196/81243 (PMC12982961; doi:10.2196/81243)
Supplement: Multimedia Appendix 4 [file jmir_v28i1e81243_app4.docx]

**Excluded learning objectives**

| **Learning objective** | **Disease** | **Content field** | **Bloom category** |
| --- | --- | --- | --- |
| The patient knows that he/she has to inform doctors about his/her allergy to Codeine and Azithromycin in  future visits. (HT 6.1) | Arterial Hypertension | Prevention of complications | Remember |
| The patient understands that this information helps prevent accidental prescription of these substances. (HT 6.2) | Arterial Hypertension | Prevention of complications | Understand |
| The patient knows that he/she has to seek medical attention if experiencing muscle pain after the dosage increase of Atorvastatin. (DM 8.1) | Diabetes mellitus | Prevention of complications | Remember |
| The patient understands that muscle pain might be a potential side effect of Atorvastatin. (DM 8.2) | Diabetes mellitus | Prevention of complications | Understand |
| The patient understands, that volume deficit can lead to kidney damage. (DKD 9.2) | Diabetic Kidney Disease | Prevention of complications | Understand |
