# Supplementary material for: Impact of GPT-4–Generated Discharge Letters on Patients’ Medical Comprehension: Prospective Crossover Study
Source: J Med Internet Res. 2026 Feb 26;28:e81243. doi: 10.2196/81243 (PMC12982961; doi:10.2196/81243)
Supplement: Multimedia Appendix 5 [file jmir_v28i1e81243_app5.docx]

Relevant fitted models were subsequently compared with a likelihood ratio (LR) test according to increasing model complexity. The LR test indicated that model m4a provided a significantly better fit than the main effects model m3 (P<.001) and at the same time described the data sufficiently well, such that a comparison to a saturated model m_sat (Letter type x Content field x Bloom category x time) did not show a significant improvement (P=.159). The model m4a_disease added the main effects of disease and did not show a significant improvement (P=.959). Notably, the repetition effect was small (P=.023, model m4a_m_time). Model m4a, which includes an interaction between Letter type and Content field as well as main effects of Bloom category and Time point was therefore selected as the best fitting model, and its estimates were used to interpret the results. All models employed a logit flexible link threshold. A complete list of model comparisons including the R script is available from the corresponding author upon reasonable request. Content field: As data in each model is restricted to one of the four content fields (Lifestyle/Disease Management, Medication, Organizational, Prevention of Complications), the variable Content Field does not appear as a statistical predictor. Fitted models were compared using a likelihood ratio (LR) test to assess the effect of the variable Letter Type. All models used a logit flexible link threshold. Medical Accuracy: Models m1 and m2 were compared with the baseline model m0 using a likelihood ratio (LR) test. The LR test indicated no effect, or only a very small effect, of Letter type and Disease type on the medical accuracy of the answers. AIC: Akaike Information Criterion; LR stat: Likelihood-Ratio Statistic; df: Degrees of Freedom; Pr(>Chisq): P-value of Chi-Square test.

**Likelihood Ratio Tests of the Relevant Cumulative Link Mixed Models**

| **Model** | **R formula** | **Number of Parameters** | **AIC** | **Log-Likelihood** | **LR stat** | **df** | **Pr(>Chisq)** |
| --- | --- | --- | --- | --- | --- | --- | --- |
| m0 | outcome ~ 1 + (1 \| participant) + (1 \| item) | 4 | 3950.0 | -1971.0 |  |  |  |
| m3 | outcome ~ letter + content + bloom + time  + (1 \| participant) + (1 \| item) | 10 | 3885.0 | -1932.5 | 77.004 | 6 | <.001 *** |
| m4a | **outcome ~ letter * content + bloom + time  + (1 \| participant) + (1 \| item)** | 13 | **3873.7** | -1923.9 | 17.330 | 3 | <.001 *** |
| m_sat | outcome ~ letter * content * bloom * time  + (1 \| participant) + (1 \| item) | 35 | 3889.2 | -1909.6 | 28.507 | 22 | .159 |
| m4a_disease | outcome ~ letter * content + bloom + time + Disease  + (1 \| participant) + (1 \| item) | 15 | 3877.6 | -1923.8 | 0.0834 | 2 | .959 |
| m4a_m_time | outcome ~ letter * content + bloom  + (1 \| participant) + (1 \| item) | 12 | 3876.9 | -1926.4 | 5.1611 | 1 | .023 * |

**Likelihood Ratio Tests of the Cumulative Link Mixed Models restricted to each Content Field**

| **Model** | **R formula** | **Number of Parameters** | **AIC** | **Log-Likelihood** | **LR stat** | **df** | **Pr(>Chisq)** |
| --- | --- | --- | --- | --- | --- | --- | --- |
| Organizat. m0 | outcome ~ bloom + time + (1 \| participant) + (1 \| item) | 6 | 559.11 | -273.56 |  |  |  |
| Organizat. m1 | outcome ~ letter + bloom + time  + (1 \| participant) + (1 \| item) | 7 | 549.21 | -267.60 | 11.908 | 1 | <.001 ** |
| Medication m0 | outcome ~ bloom + time + (1 \| participant) + (1 \| item) | 6 | 873.12 | -430.56 |  |  |  |
| Medication m1 | outcome ~ letter + bloom + time  + (1 \| participant) + (1 \| item) | 7 | 835.68 | -410.84 | 39.446 | 1 | <.001 *** |
| Prevention  m0 | outcome ~ bloom + time + (1 \| participant) + (1 \| item) | 6 | 1223.3 | -605.63 |  |  |  |
| Prevention  m1 | outcome ~ letter + bloom + time  + (1 \| participant) + (1 \| item) | 7 | 1216.3 | -601.17 | 8.9174 | 1 | <.01 ** |
| Dis.Manage.  m0 | outcome ~ bloom + time + (1 \| participant) + (1 \| item) | 6 | 1339.6 | -663.81 |  |  |  |
| Dis.Manage.  m1 | outcome ~ letter + bloom + time  + (1 \| participant) + (1 \| item) | 7 | 1340.8 | -663.42 | 0.767 | 1 | .381 |

**Likelihood Ratio Tests of the generalized Linear Mixed Effects models for Medical Accuracy**

| **Model** | **R formula** | **Number of Parameters** | **AIC** | **Log-Likelihood** | **Chisq** | **df** | **Pr(>Chisq)** |
| --- | --- | --- | --- | --- | --- | --- | --- |
| m0 | correct ~ 1 + (1 \| participant) + (1 \| item) | 3 | **525.70** | -259.85 |  |  |  |
| m1 | correct ~ letter  + (1 \| participant) + (1 \| item) | 4 | 527.34 | -259.67 | 0.3554 | 1 | .551 |
| m2 | correct ~ disease  + (1 \| participant) + (1 \| item) | 5 | 528.83 | -259.41 | 0.8707 | 2 | .647 |
