# Supplementary material for: Impact of GPT-4–Generated Discharge Letters on Patients’ Medical Comprehension: Prospective Crossover Study
Source: J Med Internet Res. 2026 Feb 26;28:e81243. doi: 10.2196/81243 (PMC12982961; doi:10.2196/81243)
Supplement: Multimedia Appendix 7 [file jmir_v28i1e81243_app7.docx]

**Number of Learning Outcomes by Letter Type**

| **Rating** | **Discharge Letter (DL)** | **Patient Letter (PL)** | **Difference  PL - DL** | **Statistics** | |
| --- | --- | --- | --- | --- | --- |
| **Overall** | | | |  |  |
| Not reported | 373 | 261 | -112 | Main effect  Letter type  P<.001 | Comparison saturated model P=.159 |
| Partially reported | 287 | 322 | 35 |  |  |
| Comprehensively reported | 413 | 490 | 77 |  |  |
| **Bloom: Remember** | | | |  |  |
| Not reported | 111 | 72 | -39 | Main effect  Bloom category  P<.001  Interaction effect  Letter type by Bloom category  P=.131 |  |
| Partially reported | 192 | 195 | 3 |  |  |
| Comprehensively reported | 242 | 278 | 36 |  |  |
| **Bloom: Understand** | | | |  |  |
| Not reported | 262 | 189 | -73 |  |  |
| Partially reported | 95 | 127 | 32 |  |  |
| Comprehensively reported | 171 | 212 | 41 |  |  |
| **Content: Organization** | | | |  |  |
| Not reported | 47 | 28 | -19 | Interaction Effect  Letter type by Content field  P<.001 |  |
| Partially reported | 49 | 52 | 3 |  |  |
| Comprehensively reported | 62 | 78 | 16 |  |  |
| **Content: Medication** | | | |  |  |
| Not reported | 64 | 16 | -48 |  |  |
| Partially reported | 61 | 68 | 7 |  |  |
| Comprehensively reported | 129 | 170 | 41 |  |  |
| **Content: Prevention of Complications** | | | |  |  |
| Not reported | 158 | 116 | -42 |  |  |
| Partially reported | 70 | 102 | 32 |  |  |
| Comprehensively reported | 83 | 93 | 10 |  |  |
| **Content: Lifestyle / Disease Management** | | | |  |  |
| Not reported | 104 | 101 | -3 |  |  |
| Partially reported | 107 | 100 | -7 |  |  |
| Comprehensively reported | 139 | 149 | 10 |  |  |
