# Supplementary material for: Impact of GPT-4–Generated Discharge Letters on Patients’ Medical Comprehension: Prospective Crossover Study
Source: J Med Internet Res. 2026 Feb 26;28:e81243. doi: 10.2196/81243 (PMC12982961; doi:10.2196/81243)
Supplement: Multimedia Appendix 8 [file jmir_v28i1e81243_app8.docx]

**Missed learning objectives**

| **Learning objective** | **Disease** | **Content field** | **Bloom category** |
| --- | --- | --- | --- |
| The patient understands that obstructive sleep apnoea might be associated with hypertension. (HT 2.2) | Arterial Hypertension | Organization | Understand |
| The patient knows that he/she has to be vigilant of symptoms such as palpitations, trembling or changes in consciousness (DM 7.1) | Diabetes mellitus | Prevention of complications | Remember |
| The patient understands that these symptoms might be signs of hypoglycaemia. (DM 7.2) | Diabetes mellitus | Prevention of complications | Understand |
| The patient understands that this information helps prevent accidental prescription of these substances. (DKD 8.2) | Diabetic Kidney Disease | Prevention of complications | Understand |
| The patient understands that regularly monitor his/her blood pressure helps MDs to adjust blood pressure therapy (DKD 10.2) | Diabetic Kidney Disease | Lifestyle/Disease Management | Understand |
| The patient understands the significance of adhering to a Mediterranean diet as a means to attain the desired weight loss. (DKD 12.2) | Diabetic Kidney Disease | Lifestyle/Disease Management | Understand |
